# Pure Total Flavonoids From Citrus Protect Against Nonsteroidal Anti-inflammatory Drug-Induced Small Intestine Injury by Promoting Autophagy *in vivo* and *in vitro*

**DOI:** 10.3389/fphar.2021.622744

**Published:** 2021-04-19

**Authors:** Shanshan Chen, Jianping Jiang, Guanqun Chao, Xiaojie Hong, Haijun Cao, Shuo Zhang

**Affiliations:** ^1^ First Affiliated Hospital, Zhejiang Chinese Medical University, Zhejiang, China; ^2^ Department of Pharmacy, School of Medicine, Zhejiang University City College, Zhejiang, China; ^3^ Zhejiang You-du Biotech Limited Company, Quzhou, China; ^4^ Sir Run Run Shaw Hospital, Zhejiang, China

**Keywords:** autophagy, pure total flavonoids from citrus, non-steroidal anti-inflammatory drugs, PI3K-AKT pathway, small intestine injury

## Abstract

Small intestine injury is an adverse effect of non-steroidal anti-inflammatory drugs (NSAIDs) that urgently needs to be addressed for their safe application. Although pure total flavonoids from citrus (PTFC) have been marketed for the treatment of digestive diseases, their effects on small intestine injury and the underlying mechanism of action remain unknown. This study aimed to investigate the potential role of autophagy in the mechanism of NSAID (diclofenac)-induced intestinal injury *in vivo* and *in vitro* and to demonstrate the protective effects of PTFC against NSAID-induced small intestine disease. The results of qRT-PCR, western blotting, and immunohistochemistry showed that the expression levels of autophagy-related 5 (Atg5), light chain 3 (LC3)-II, and tight junction (TJ) proteins ZO-1, claudin-1, and occludin were decreased in rats with NSAID-induced small intestine injury and diclofenac-treated IEC-6 cells compared with the control groups. In the PTFC group, Atg5 and LC3-II expression, TJ protein expression, and the LC3-II/LC3-I ratio increased. Furthermore, the mechanism by which PTFC promotes autophagy *in vivo* and *in vitro* was evaluated by western blotting. Expression levels of p-PI3K and p-Akt increased in the intestine disease-induced rat model group compared with the control, but decreased in the PTFC group. Autophagy of IEC-6 cells was upregulated after treatment with a PI3K inhibitor, and the upregulation was significantly more after PTFC treatment, suggesting PTFC promoted autophagy through the PI3K/Akt signaling pathway. In conclusion, PTFC protected intestinal barrier integrity by promoting autophagy, which demonstrates its potential as a therapeutic candidate for NSAID-induced small intestine injury.

## Introduction

Non-steroidal anti-inflammatory drugs (NSAIDs), such as diclofenac and aspirin, are the most prescribed drugs worldwide and are commonly used in the treatment of chronic pain, tumor chemoprevention, and cardio-cerebrovascular diseases (Gwee et al., 2018). The expanding use of capsule endoscopy and double-balloon enteroscopy have increased the incidence of adverse effects on the digestive tract, including small intestine injury, that is currently receiving increased attention. A recent study confirmed that the incidence of NSAID-induced lower digestive tract injury is higher than that of the upper digestive tract (Sostres et al., 2013). Injury to the small intestine induced by NSAIDs is characterized by intestinal mucosal erosion, ulceration, stricture, and even bleeding, which can be severely harmful to the health of the individual (Shin et al., 2017). Therefore, small intestine injury is an adverse event associated with NSAID treatment that urgently needs to be addressed to allow for the safe administration of these drugs.

Dysfunction of the mechanical barrier of the intestinal mucosa is considered to play a vital role in the occurrence and development of NSAID-induced lower digestive tract injury (Bjarnason et al., 2018). Furthermore, intestinal mucosa permeability increases in NSAID-induced enteropathy, which causes intestinal bacteria, toxins, bile acids, and proteolytic enzymes to penetrate intestinal epithelial cells and cause intestinal mucosal erosion and ulceration, leading to a series of symptoms (Ravikumar et al., 2004). However, the mechanism by which the intestinal mucosa mechanical barrier is impaired has not been clarified. Elucidating its regulatory mechanism would facilitate the identification of effective strategies to prevent and treat NSAID-induced intestinal injury. Previous studies have determined that expression of intestinal tight junction (TJ) proteins, including zonula occludens-1 (ZO-1), claudin-1 (CLDN-1), and occludin (OCLN), and cytoskeleton-related proteins decreases in NSAID-induced small intestine injury. Destruction of the intestinal epithelial cytoskeleton and TJs increases intestinal epithelium permeability, which in turn causes intestinal mucosa damage (Chao and Zhang, 2012).

A recent study showed that autophagy is closely involved in the barrier function of the intestinal mucosa and regulation of intestinal epithelial TJs (Wong et al., 2019). Autophagy is generally considered a process by which cells are renewed. It degrades senescent organelles, long-lived proteins, and invading pathogens through lysosomes and recycles the degradation products to maintain the physiological processes required for basic life activities (Mizushima and Komatsu, 2011; Galluzzi et al., 2014). Recent studies have found that autophagy is affected in various diseases by the regulation of AMP-activated protein kinase (AMPK), mitogen-activated protein kinase (MAPK), Beclin-1/B-cell lymphoma (Bcl)-2, and the mammalian target of rapamycin (mTOR) pathway (Mohammadinejad et al., 2019). However, the mechanism underlying the intestinal mucosal mechanical barrier impairment has not been clarified.

Citrus flavonoids have been studied in recent years owing to their beneficial characteristics, such as anti-oxidative, anti-inflammatory, and pro-cardiovascular activities (Bondonno et al., 2018; Vazhappilly et al., 2019). Pure total flavonoids from citrus (PTFC) are flavonoids isolated and purified from the dry and ripe peels of citrus species (Citrus Changshan-huyou Y.B Chang; Qu Zhi Qiao), which are mainly produced in Changshan and Quzhou in Zhejiang Province, China. PTFC consist of four flavonoids, naringin, neohesperidin, narirutin, and hesperidin. Naringin plays an antioxidant role in improving liver injury and downregulates inflammatory mediators (Adil et al., 2015). *In vitro* studies have shown that naringin decreases hepatic stellate cell activity and inhibits liver fibrosis by inhibiting mTOR-autophagy (Shi et al., 2016). Narirutin inhibits the production of inflammatory mediators via NF-kB and MAPKs in lipopolysaccharide (LPS)-stimulated macrophages (Ha et al., 2012). In addition, naringin can increase the level of antioxidants *in vivo* to protect the liver and small intestine from oxidative damage caused by free radicals (Wu et al., 2017). To date, only a few studies have investigated the potential implication of citrus flavonoids in intestinal mucosal barrier repair (Stevens et al., 2019), making further investigation needed. Furthermore, the effects of PTFC on autophagy remain unknown. To elucidate this important interaction, we investigated whether improvement in NSAID-induced small intestine injury by PTFC is mediated by its promotion of autophagy.

## Materials and Methods

### PTFC Extraction and Purification

PTFC were prepared as previously described (Jiang et al., 2019). Briefly, 1,000 g of Qu Zhi Qiao (*Citrus Paradisi cv. Changshanhuyou*) fruit peel was extracted twice with 0.10% calcium hydroxide [Ca(OH)_2_] solution at 100°C for 1.5 h. Filtrates were mixed and decompressed. The total flavonoid preparations were at a concentration of 3.83 mg/ml. The total flavonoids were separated and enriched using HPD-300 macroporous resin. The amounts of loaded samples and the method of eluting the samples were as described above. The eluant was collected and concentrated by drying for use in subsequent experiments.

### PTFC Flavonoid Content

The PTFC composition was analyzed by high-performance liquid chromatography (HPLC) using a system that included a quaternary gradient pump, an online degasser, a UV detector, and a column thermostat produced by Shimadzu Corporation. Chromatographic separation was performed at 25°C on a Hypersil SB C18 column (Thermo Fisher Scientific, MA, United States). The mobile phase was water-acetonitrile and the samples were eluted using a gradient method consisting of the following elution gradient: 0–15 min, 20% acetonitrile; 15–35 min, 60–100% acetonitrile; 42–45 min, 100–20% acetonitrile; 45–50 min, 20% acetonitrile. The flow rate of the mobile phase was 1.0 ml/min, the wavelength was 283 nm, and the injection volume was 10 μl. The flavonoids in the samples were identified based on the chromatographic peaks of the standard substances that constitute PTFC (neohesperidin, naringin, narirutin, and hesperidin).

The total flavonoid content of PTFC was determined as previously described (Jiang et al., 2019). Briefly, each flavonoid extract was dissolved in methanol (1:1, w/v). Then, 0.5 ml of 10% Al(NO_3_)_3_ solution was added followed by 0.5 ml of 5% NaNO^2^ solution. The absorbance of the samples was measured at 0, 5, and 10 min at a wavelength of 510 nm. The total flavonoid content in the PTFC was calculated using a standard curve.

### Animals

Eight-week-old male Sprague–Dawley rats (220 ± 20 g) were obtained from Zhejiang Chinese Medical University and were fed standard laboratory chow and provided tap water. The rats were randomly divided into three groups and housed in cages (four rats per cage) in climate-controlled rooms at 20 ± 2°C and 50–60% humidity on a 12 h light/dark cycle. The study was approved by the local Animal Ethics Committee of Zhejiang Chinese Medical University (Zhang et al., 2019).

### Diclofenac-Induced Small Intestine Injury and Treatments

Intestinal injury was induced using diclofenac based on a method previously developed in our laboratory (Chao and Zhang, 2012). The treatment course and dose of diclofenac were chosen as it causes small intestine injury similar to those induced by NSAIDs in humans. To establish the experimental model, non-fasted rats (*n* = 8 per group) were treated by intragastric administration of diclofenac (7.5 mg/kg) twice a day for 5 days. The control groups were treated with intragastric administration of 2 ml of saline twice a day for 5 days. PTFC (100 mg/kg/day), which was supplied by Zhejiang Chinese Medical University, was administered 9 days before the initial diclofenac administration and then both drugs were co-administered on the final 5 days. All rats were anesthetized by an intraperitoneal injection of 50 mg/kg sodium pentobarbital after the drug treatment, the small intestines were harvested for observation, and the tissue prepared for subsequent experiments.

### Cell Culture and Treatments

IEC-6 cells were purchased from American Type Culture Collection (ATCC, Manassas, VA, United States) and cultured in Dulbecco's Modified Eagle Medium (DMEM), high glucose medium supplemented with penicillin (100 U/ml), streptomycin (100 mg/L), 10% fetal bovine serum (FBS), and insulin (0.1 U/ml). The cells maintained in a 37°C incubator containing 5% CO_2_. The experiments consisted of the four following groups:

Model group: When the cell confluence reached approximately 60%, diclofenac sodium was added to each well at a final concentration of 60 μM. After 48 h of induction, the cells were collected for western blot analysis and immunofluorescence staining.

PTFC group: During the second 24 h of the 48 h cell modeling period, PTFC were added for continuous treatment at a final concentration of 1 mg/ml.

PI3K inhibition group: During the second 24 h of the 48 h cell modeling period, PI3K inhibitor LY294002 was added for continuous treatment at a final concentration of 20 μM.

PTFC + PI3K inhibition group: During the second 24 h of the 48 h cell modeling period, both the PI3K inhibitor LY294002 (final concentration of 20 μM) and PTFC (final concentration of 1 mg/ml) were added.

### General Tissue Examination

General examination of the small intestine was performed on a 10 cm portion dissected from the ileocecal region. Injury to the small intestine tissue was evaluated and the ulcer index determined based on scoring of ulcer measurements using vernier calipers. The Reuter score was also used to quantify the intestinal damage according to the degree of adhesion (Endo et al., 2010). The total score of intestinal damage was the sum of both measurements (Table 1).

**TABLE 1:** Ulcer score and Reuter score determination.

Reuter score		
i) Ulcer scores	0	No ulceration occurred
	1	Local hyperemia, no ulceration
	2	Ulceration without congestion or bowel wall thickening
	3	Ulceration and inflammation in one area
	4	Two or more areas of ulcer with inflammation <1 cm
	5	Several ulcers ≥1 cm and inflammation
ii) adhesion score	0	No adhesion
	1	Light adhesion, slight force required to separate the colon from other tissues
	2	Heavy adhesion

### RNA Extraction and Real-Time Reverse Transcription Polymerase Chain Reaction (qRT-PCR) Analysis

Intestinal tissues and IEC-6 cells were collected and total RNA extracted using a TRIzol^®^ Plus RNA Purification Kit (Thermo Fisher Scientific) according to the manufacturer’s protocol. Complementary DNA (cDNA) was synthesized from the extracted RNA using SuperScript III First-Strand Synthesis SuperMix (Thermo Fisher Scientific) following the manufacturer’s instructions. Real-time PCR was performed using PowerUp™ SYBR™ Green Master Mix (Applied Biosystems, United States) according to the manufacturer’s instructions. The cycling conditions used were 95°C for 2 min, followed by 40 cycles of amplification at 95°C for 15 s and 60°C for 1 min. Relative expression of the target genes was normalized to glyceraldehyde 3-phosphate dehydrogenase (GAPDH) expression, evaluated using the 2^−∆∆Ct^ method (Livak and Schmittgen, 2001), and expressed as a ratio to the control values in the experiment. The PCR rat primer sequences used in this study are listed in Table 2.

**TABLE 2:** Polymerase chain reaction (PCR) primers.

Gene	GenBank accession	Primer sequences (5–3′)	Amplicon size (bp)
*GAPDH*	NM_017008.4	F: AAG​GTC​GGT​GTG​AAC​GGA​TTT​G	127
		R: CAT​GTA​GAC​CAT​GTA​GTT​GAG​GTC​A	
*Atg5*	NM_001,014,250.1	F: TCA​GCT​CTG​CCT​TGG​AAC​ATC​A	95
		R: AAG​TGA​GCC​TCA​ACT​GCA​TCC​TT	
*Z O -1*	NM_001,106,266.1	F: GAC​CCT​GAC​CCA​GTG​TCT​GAT​AA	119
		R: CTA​TCC​CTT​GCC​CAG​CTC​TTC​T	
*CLDN 1*	NM_031699.2	F: GGA​TGG​ATC​GGC​TCT​ATC​GTC​A	90
		R: GAT​GGC​CTG​AGC​AGT​CAC​GAT	
*OCLN*	NM_031329	F: CCA​ACG​GCA​AAG​TGA​ATG​GCA​AGA	105
		R: CCA​CGG​ACA​AGG​TCA​GAG​GAA​TCT	

GAPDH, glyceraldehyde 3-phosphate dehydrogenase; Atg5, autophagy-related 5; ZO-1, zonula occludens; CLDN-1, claudin-1; OCLN, occludin; F, forward; R, reverse; bp, base pair.

### Western Blot Analysis

Proteins from the small intestine tissue samples and IEC-6 cells were extracted using radioimmunoprecipitation assay (RIPA) buffer (Thermo Fisher Scientific) supplemented with a protease and phosphatase inhibitor cocktail (Thermo Fisher Scientific) and then quantified using a BCA protein assay kit (Beyotime Biotechnology, China). Approximately 50 μg of protein was loaded onto each lane of an acrylamide gel and separated by sodium dodecyl sulfate-polyacrylamide gel electrophoresis (SDS-PAGE). The electrophoretically separated protein bands were transferred onto a Hybond-P polyvinylidene fluoride (PVDF) membrane (GE Healthcare, United States). After blocking with 5% non-fat milk in Tris-buffered saline containing 0.1% Tween-20, the membranes were incubated at 4°C overnight with the following primary antibodies: rabbit anti-light chain 3 (LC3, 1:1,000, Cell Signaling Technology, United States), rabbit anti-ZO1 (1:500, Abcam, United Sttaes), rabbit anti-CLDN1 (1:500 and 1:1,000, Abcam), rabbit anti-OCLN (1:1,000, Abcam), rabbit anti-PI3K (1:500, Abcam), rabbit anti-PI3K p85 alpha (phospho Y607, 1:500, Abcam), rabbit anti-Akt (1:1,000, Cell Signaling Technology), rabbit anti-Akt (phosphor Ser473, 1:1,000, Cell Signaling Technology), rabbit anti-mTOR (1:1,000, Cell Signaling Technology), or rabbit anti-mTOR (phosphor Ser2448, 1:1,000, Cell Signaling Technology). Rabbit anti-GAPDH (1:10,000, Abcam) was used as an internal control. The membranes were then washed and incubated with a horseradish peroxidase (HRP)-labeled goat anti-rabbit secondary antibody (1:5,000, Thermo Fisher Scientific). The HRP was detected using the luminol-based enhanced chemiluminescent SuperSignal West Dura Extended Duration Substrate (Thermo Fisher Scientific). Protein expression was visualized using X-ray film and the band intensities were quantitated using Image Pro Plus 6.0 software (Media Cybernetics, United States). Data are presented as ratios of the optical density (OD) of the target protein band to that of the GAPDH band. For the p-PI3K, p-Akt, and p-mTOR subunits, the data are presented as ratios of the OD of phosphorylated bands to that of the total subunit band.

### Histopathology and Histological Examination

The small intestine tissue samples were fixed with 10% formalin in phosphate-buffered saline (PBS), embedded in paraffin, cut as 3 μm-thick sections, mounted onto microscope slides, and stained with hematoxylin and eosin (H&E) for histological examination according to standard techniques. The stained sections were then examined using a light microscope (Olympus, Japan) to histologically evaluate the small intestine injuries.

### Immunohistochemical (IHC) Assay

Paraffin-embedded small intestine tissue sections (5 μm) were deparaffinized in xylene and then rehydrated through a graded series of ethanol and distilled water. The rehydrated tissue sections were microwaved for antigen retrieval, treated with 3% hydrogen peroxide (H_2_O_2_) for 5 min, blocked with 5% bovine serum albumin. The blocked tissue sections were incubated at 4°C overnight with the following antibodies: rabbit anti-LC3 (1:1,000, Cell Signaling Technology), rabbit anti-ZO 1 (1:75, Santa Cruz, United States), rabbit anti-OCLN (1:100, Abcam), and rabbit anti-CLDN1 (1:200, Abcam). The antibody binding to their respective target antigens were detected using an Envision™ Detection Kit (DAKO, Denmark). All sections were counterstained with hematoxylin and examined under an light microscope. In addition, quantitative IHC analysis was performed using Image Pro Plus 6.0 software (Media Cybernetics). Data are shown as the fold-change of the average integrated OD (IOD) per area.

### Immunofluorescence Staining

IEC6 cells (3 × 10^4^) were seeded onto glass coverslips in 12-well plates and cultured overnight at 37°C in a 5% CO_2_ incubator. After treatment with diclofenac sodium and PTFC, the cells were fixed with 4% paraformaldehyde for 30 min, followed by three PBS washes. The cells were subsequently permeabilized with 0.5% Triton X-100 solution for 5 min, blocked with 10% normal donkey serum for 1 h at room temperature. The cells were then incubated at 4°C overnight with primary antibody rabbit anti-LC3 (1:200, Cell Signaling Technology). After three washes with PBS, the cells were incubated for 1 h with Alexa Fluor® 488 Donkey Anti-Rabbit IgG (1:200; Jackson ImmunoResearch, United States). Finally, the cells were counterstained with DAPI (Beyotime, China) for 5 min and the coverslips were mounted in 10 μl of FluroGuard anti-fade solution (Bio-Rad, United States). The positive control group of cells were treated with chloroquine at a final concentration of 60 μM for 48 h.

### Statistical Analysis

All data were analyzed using the Statistical Package for the Social Sciences (SPSS) 17.0 software and expressed as mean ± standard error of the mean (SEM). Comparisons between two groups were performed using a two-tailed *t*-test. Differences between multiple groups were assessed by one-way analysis of variance (ANOVA). Test results showing *p* < 0.05 (*) and *p* < 0.01 (**) were considered statistically significant.

## Results

### PTFC Total Flavonoid Content

**FIGURE 1:**
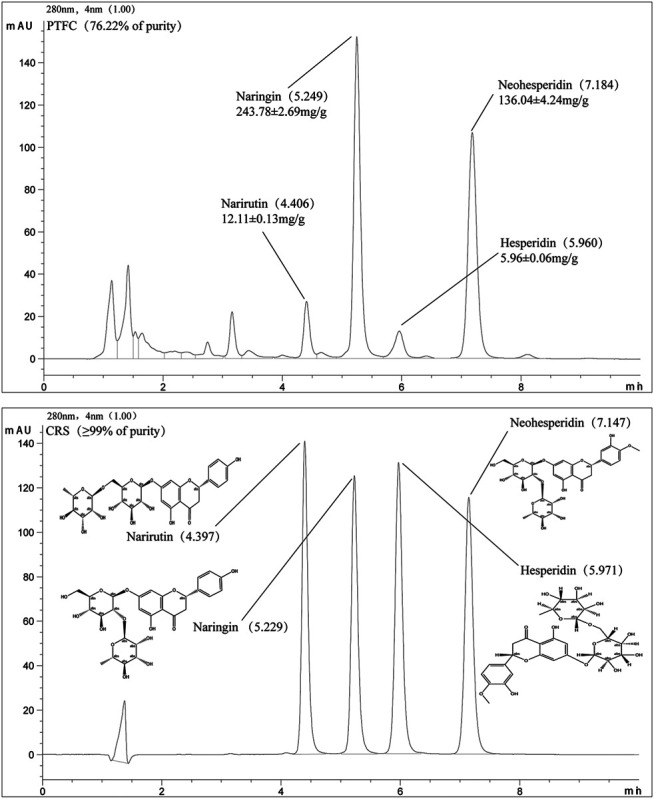
HPLC chromatographic profile of PTFC and standard flavanones narirutin, naringin, neohesperidin, and hesperidin.

The HPLC chromatogram profile of PTFC is shown in Figure 1. The HPLC chromate-graphic profile revealed that narirutin, naringin, neohesperidin, and hesperidin were present in the PTFC sample, with retention time peaks of 4.397, 5.229, 5.971, and 7.147 min, respectively. The total flavonoid content (purity) of PTFC as rutin equivalents was 76.22%. Based on the standard curve, the narirutin, naringin, neohesperidin, and hesperidin content in PTFC were 12.11 ± 0.12 mg/g, 243.78 ± 2.69 mg/g, 5.96 ± 0.06 mg/g, and 136.04 ± 4.24 mg/g, respectively.

### *In vivo* Inhibition of Autophagy and Down Regulation of TJ-Related Proteins in the Diclofenac-Treated NSAID Model Group

**FIGURE 2:**
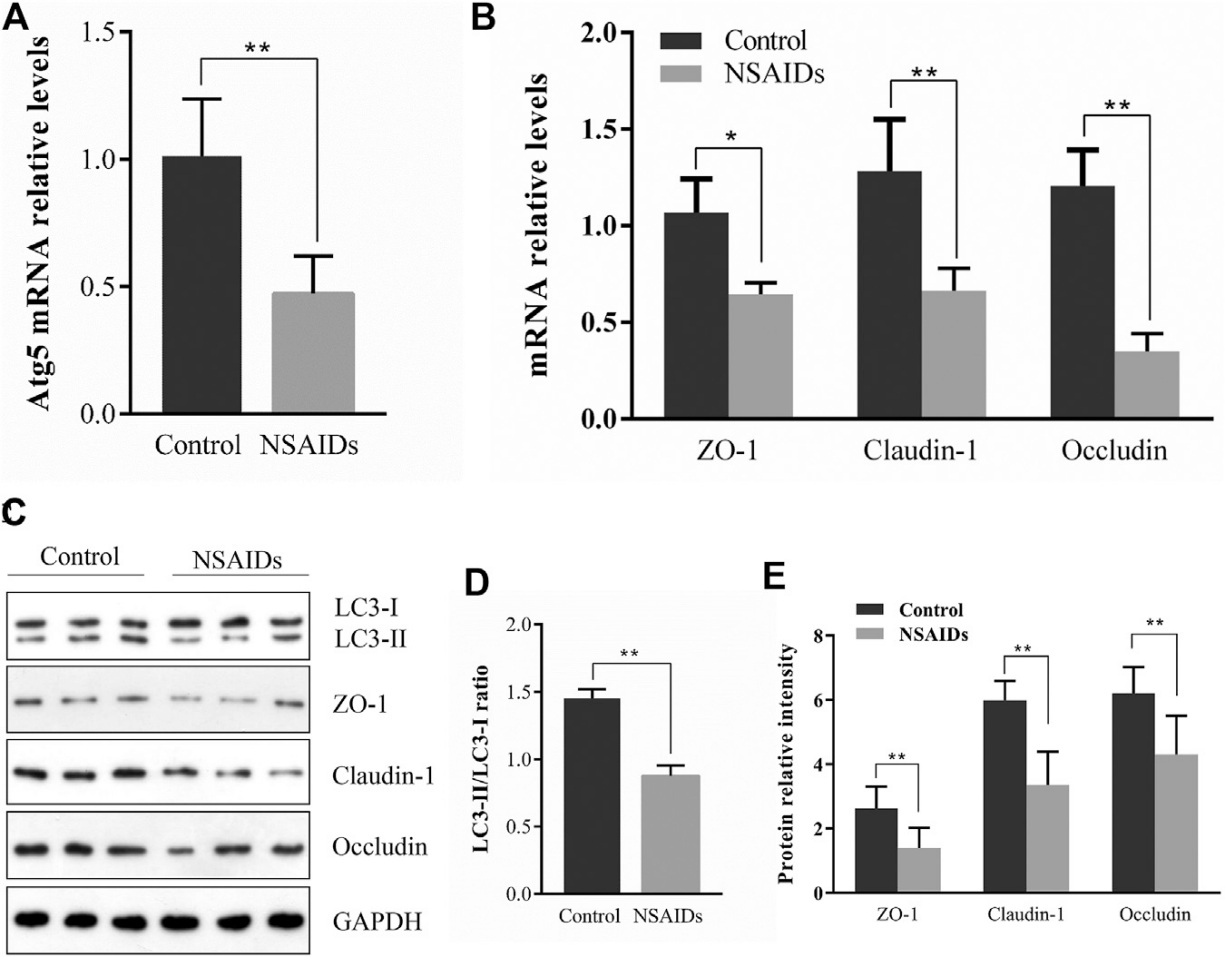
Relationship between autophagy and tight junction (TJ) barrier in nonsteroidal anti-inflammatory drug (NSAID)-induced small intestine injury. Levels of mRNA were determined in the NSAID model group and control group using quantitative reverse transcription-polymerase chain reaction (qRT-PCR) for **(A)** autophagy-related 5 (Atg5) and **(B)** zonula occludens (ZO-1), claudin (CLDN)-1, and occludin (OCLN) **(C)** Glyceraldehyde 3-phosphate dehydrogenase (GAPDH) was used as a loading control (**D,E)** Western blotting was used to analyze protein levels in the NSAID model group and control group **(D)** Protein levels of light chain 3 (LC3) forms LC3-II and LC3-I were determined using ImageJ software and the signal density ratios of LC3-II/LC3-I were calculated **(E)** Protein levels of ZO-1, CLDN-1, and OCLN. **p < 0.01.

According to qRT-PCR analysis of mRNA levels, Atg5 expression in the diclofenac-treated NSAID rat model group with enhanced small intestine injury was lower than that in the untreated (uninjured) control group (Figure 2A). Meanwhile, qRT-PCR and western blot analysis of ZO-1, CLDN-1, and OCLN showed that expression levels of these major TJ-related proteins were lower in the NSAID model group than that in the control group (Figures 2B,E). Western blot analysis of the autophagy marker LC3-II showed that the NSAID model group exhibited significantly lower LC3-II protein levels than those of the control group, and the LC3-II/LC3I ratio was also lower (Figure 2D).

### PTFC Increased TJ-Related Protein Expression and Inhibited NSAID-Induced Small Intestine Injury Progression *in vivo* and *in vitro*

**FIGURE 3:**
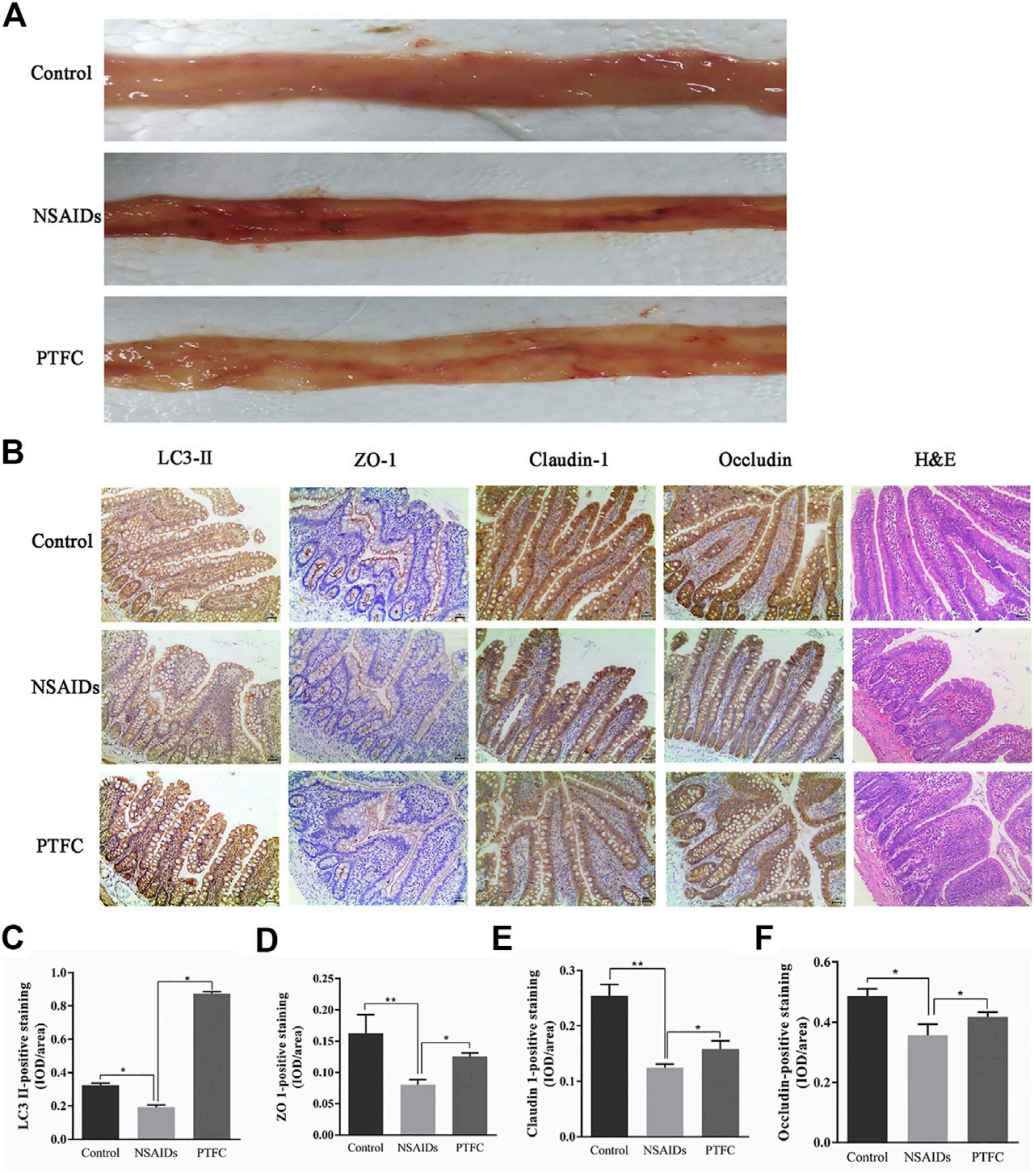
Morphological examination of various experimental groups or rats **(A)** Morphological changes in the intestinal mucous membrane were observed **(B)** Hematoxylin and eosin (H&E) staining revealed severe injuries in the small intestines of rats in NSAID model group, whereas the PTFC group showed significant improvement in bowel lesions (×200 magnification). Immunohistochemistry (IHC) was used to evaluate expression of **(C)** light chain 3 (LC3)-II **(D)** zonula occludens (ZO)-1 **(E)** claudin (CLDN)-1, and **(F)** occludin (OCLN) in control, NSAID model, and PTFC groups of rats.

**FIGURE 4:**
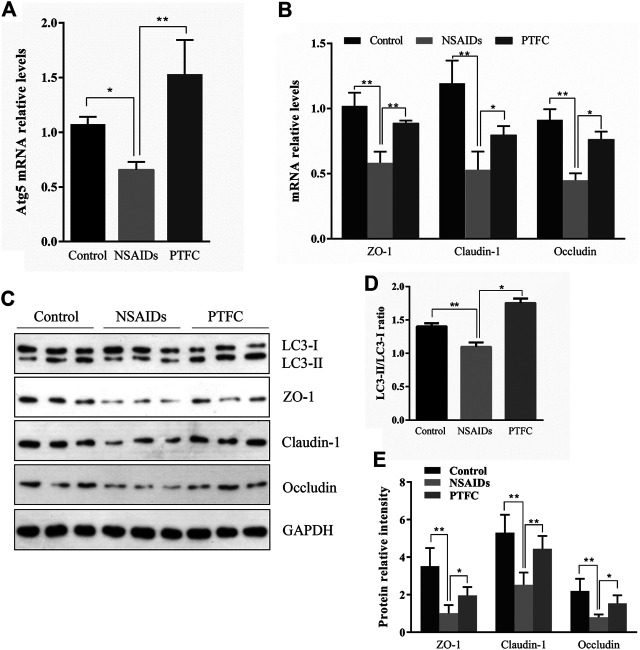
PTFC increases TJ-related protein levels and inhibits NSAID-induced small intestine injury progression in rats. Levels of mRNA were evaluated in the NSAID model group, PTFC group, and control group of rats using quantitative reverse transcription-polymerase chain reaction (qRT-PCR) for **(A)** autophagy-related 5 (Atg5) and **(B)** ZO-1, CLDN1, and OCLN **(C)** Western blot analysis was used to determine protein expression levels in the groups of rats **(D)** Levels of light chain 3 (LC3) were determined and the signal intensity ratio of LC3-II/LC3-I was calculated using ImageJ software (**p < 0.01) **(E)** Protein expression of ZO-1, CLDN1, and OCLN were measured in all groups using western blotting (*p < 0.05 and **p < 0.01).

**FIGURE 5:**
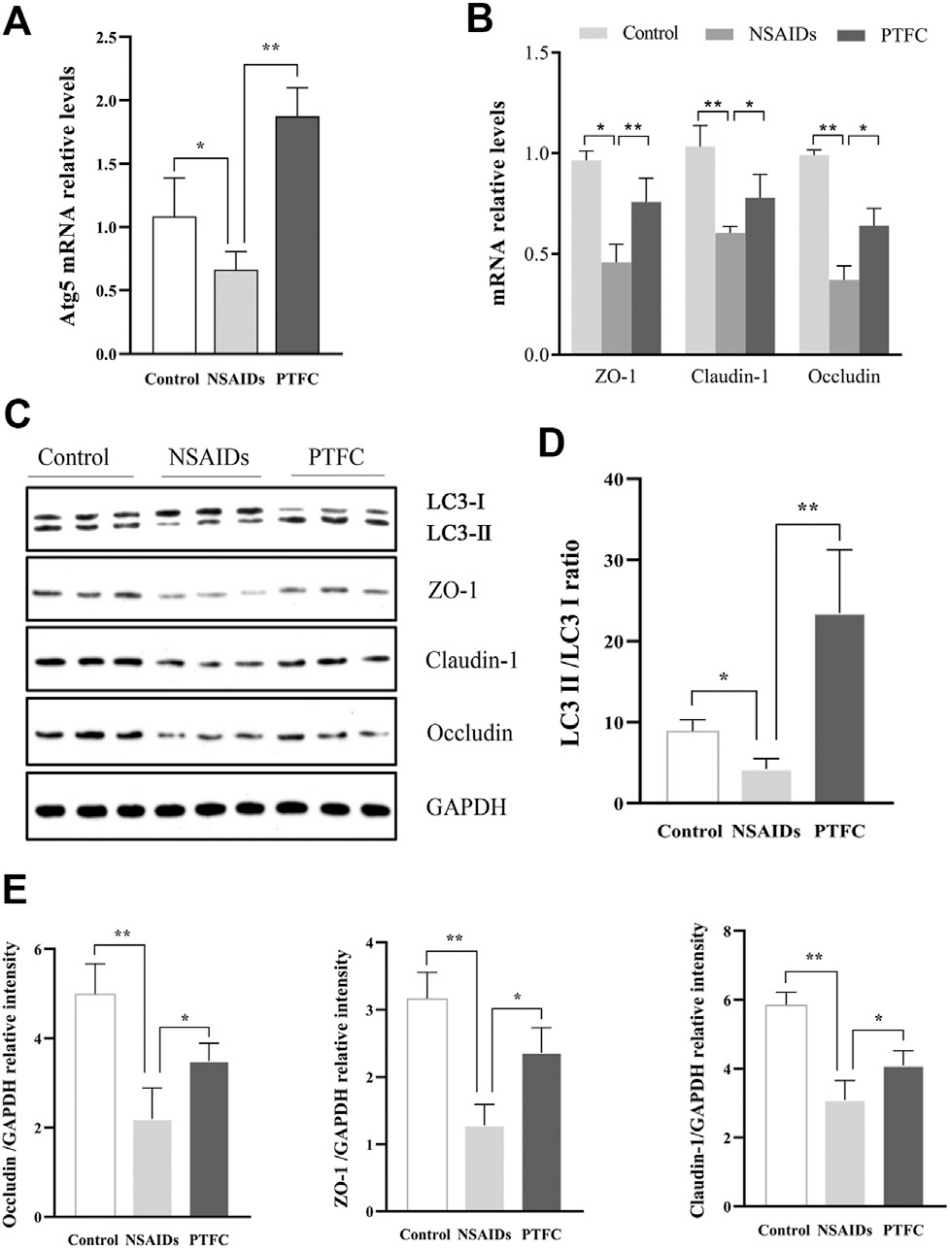
PTFC promotes autophagy and increases TJ-related protein levels in IEC-6 cells. IEC-6 cells were untreated for the 48 h of culture (Control), treated with diclofenac for the 48 h of culture (NSAID), or treated with diclofenac for the 48 h of culture plus treatment with PTFC for the final 24 h or culture (PTFC). The mRNA levels were then detected in all groups using quantitative reverse transcription-polymerase chain reaction (qRT-PCR) **(A)** mRNA levels of autophagy-related 5 (Atg5) **(B)** mRNA levels of ZO-1, CLDN1, and OCLN **(C)** Western blot analysis was used to determine protein expression levels in the groups of cells **(D)** Levels of light chain 3 (LC3) were determined and the signal intensity ratio of LC3-II/LC3-I density ratio was calculated using ImageJ software (**p < 0.01) **(E)** Protein expression of ZO-1, CLDN1, and OCLN was measured in all groups using western blotting (*p < 0.05 and **p < 0.01).

General tissue examination revealed that in the experimentally established NSAID-induced rat model, the mucosa was congested, edematous, eroded, and even ulcerated (Figure 3A and Table 3). In contrast, the PTFC group showed only mild hyperemia and edema. H&E staining showed that the small intestines of rats in the diclofenac-treated NSAID model group were severely injured with marked atrophy of the villi, irregular arrangement of glands, and considerable inflammatory cell infiltration. In contrast, the PTFC group exhibited significant improvements in small bowel lesions. IHC analysis revealed that LC3 expression levels were highest in the cytoplasm (Figure 3B). The LC3 signal showed low levels of cytoplasmic staining in the NSAID-induced model group, whereas strong staining was observed in the PTFC group. The IHC signal intensities for TJ proteins were consistent among groups. In the small intestines of rats and IEC-6 cells, expression levels of TJ proteins ZO-1, CLDN-1, and OCLN were decreased in the model group compared to that in the control group according to both qRT-PCR and western blotting, and they exhibited a greater degree of small intestine injury. Administration of PTFC attenuated the decrease in TJ protein expression and was associated with intestinal mucosal barrier repair in the NSAID-induced small intestine injury model (Figures 4B,E; Figures 5B,E).

**TABLE 3:** Macroscopic examination of small intestine injury in rats.

Experimental groups (n = 8 per group)	Reuter score
Control (A)	0.00 ± 0.00
Model (B)	5.25 ± 0.886^a^ ^,^ ^b^
PTFC (C)	1.125 ± 0.641^a^ ^,^ ^c^

^a^ *p* < 0.05, compared with the control (A) group.

^b^ *p* < 0.05, compared with PTFC (C) groups.

^c^ *p* < 0.05, compared with model (B) group.

QRT-PCR results revealed that Atg5 mRNA levels in the PTFC group were higher than those in the control group (Figure 4A; Figure 5A), whereas levels in the diclofenac-treated NSAID model group were lower. Western blot analysis of LC3-II protein expression in all three groups showed that the PTFC group exhibited a significant increase in LC3-II protein levels and the LC3-II/LC3-I ratio, whereas the diclofenac-treated NSAID model group exhibited decreased values (Figure 4D; Figure 5D).

### PTFC Treatment Increased Autophagosome Formation *in vitro*

**FIGURE 6:**
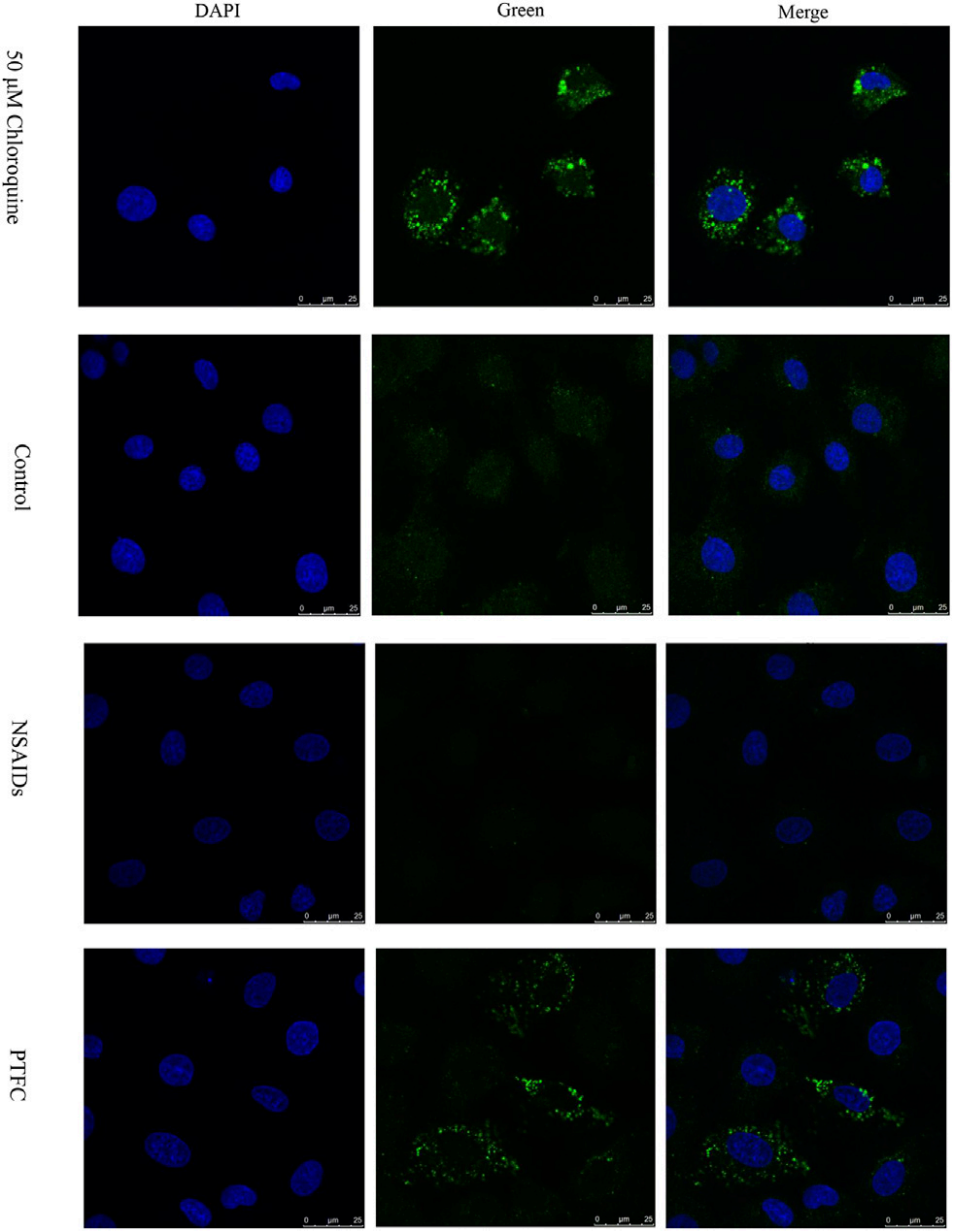
Effect of PTFC on the formation of autophagosome in IEC-6 cells. Immunofluorescence staining of LC3-II (Green) was performed. Nuclei were stained with DAPI. Chloroquine (50 μM) was used as positive control. The results revealed that the LC3-II signal in the diclofenac-treated model group (NSAIDs) was significantly decreased compared with that in the untreated group (Control), but the positive signal in the PTFC treatment group (PTFC) was significantly enhanced.

In order to evaluate the effect of PTFC on the formation of autophagosomes, immunofluorescence staining for LC3-II was performed in IEC-6 cells (Figure 6). The results showed the LC3-II signal in the diclofenac-treated model group was significantly decreased compared with that in the control group. Furthermore, the positive signal in the PTFC treatment group was significantly enhanced.

### PTFC Increased Autophagy Through PI3K/Akt/mTOR Signaling *in vivo* and *in vitro*

**FIGURE 7:**
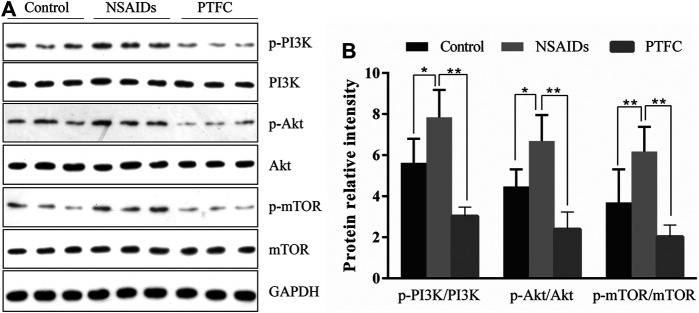
PTFC increases autophagy in rats through PI3K/Akt Signaling **(A)** Western blot analysis was performed to determine expression levels of phosphoinositide 3-kinase (PI3K), phosphorylated-PI3K (p-PI3K), Akt, phosphorylated-Akt (p-Akt), mechanistic target of rapamycin (mTOR), and phosphorylated mTOR (p-mTOR) in the NSAID model group, PTFC group, and Control group of rats **(B)** Density ratios of phosphorylated to unphosphorylated proteins (p-PI3K/PI3K, p-Akt/Akt, and p-mTOR/mTOR) were calculated using ImageJ software. Glyceraldehyde 3-phosphate dehydrogenase (GAPDH) was used as a loading control (*p < 0.05 and **p < 0.01).

**FIGURE 8:**
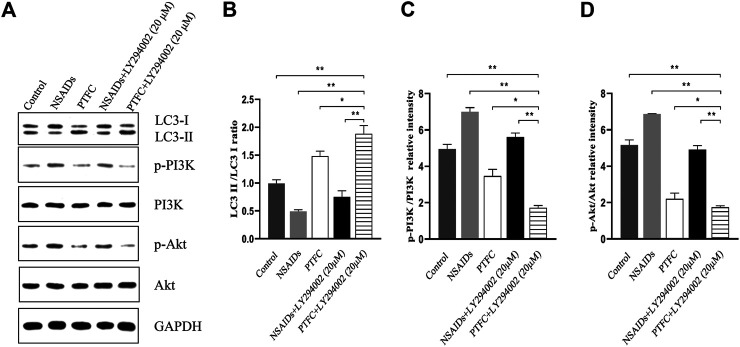
PTFC increases autophagy through PI3K/Akt Signaling in IEC-6 cells **(A)** Western blot analysis was performed to determine expression levels of levels of light chain 3 (LC3), phosphoinositide 3-kinase (PI3K), phosphorylated-PI3K (p-PI3K), Akt, and phosphorylated-Akt (p-Akt) **(B–D)** Density ratios of LC3-II/LC3-I and phosphorylated/unphosphorylated proteins (p-PI3K/PI3K, p-Akt/Akt) were calculated using ImageJ software. Glyceraldehyde 3-phosphate dehydrogenase (GAPDH) was used as a loading control (*p < 0.05 and **p < 0.01).

Expression levels of p-PI3K, PI3K, p-Akt, Akt, p-mTOR, and mTOR were determined in the small intestine of rats by western blot analysis (Figure 7A). In addition, the ratios of phosphorylated to unphosphorylated proteins (p-PI3K/PI3K, p-Akt/Akt, and p-mTOR/mTOR) were calculated for all groups (Figure 7B). The results revealed higher values in the NSAID model groups and lower values in the PTFC groups compared to those in the control group. The ratios of phosphorylated to unphosphorylated proteins (p-PI3K/PI3K and p-Akt/Akt) in IEC-6 cells treated with the PI3K inhibitor LY294002 and PTFC were significantly lower than those in the NSAID model group (Figures 8C,D). The PTFC + PI3K inhibitor group demonstrated the most significant decrease. Phosphorylation of LC3-II showed the opposite trend (Figure 8B). This suggested that PTFC promotes autophagy through PI3K/Akt signaling.

## Discussion

In the present study, we used a diclofenac-induced small intestine injury rat model to explore the role of autophagy in the regulation of intestinal mucosal barrier repair by determining the expression levels of LC3-II and Atg5. The induction of autophagy may protect the intestinal mucosal barrier. LC3-II expressed on the autophagosome membrane is a classic marker of autophagy, and the presence of LC3 in autophagosomes and the conversion of LC3-I to LC3-II are considered indicators of autophagy (Sakiyama et al., 2009; Yu et al., 2013; Zhu et al., 2015). Furthermore, among the Atg proteins, Atg5 is indispensable for autophagic vesicles and its knockout inhibits autophagy, suggesting that it has a central role in autophagy (Saha et al., 2018). Our results demonstrated lower expression levels of Atg5, LC3-II, and TJ proteins ZO-1, CLDN-1, and OCLN, as well as lower LC3-II/LC3-I ratios in the NSAID-induced small intestine injury rat model group compared to those in the control group. This indicated that autophagy may have affected TJ protein expression in NSAID-induced intestinal injury.

Understanding how NSAIDs modulate autophagy will help elucidate the role of autophagy in intestinal homeostasis and NSAID enteropathy. Recent reports suggest that autophagy in various diseases is affected by the regulation of the AMPK, MAPK, MAPK, Beclin-1, Bcl-2, and mTOR pathways (Mohammadinejad et al., 2019). The serine/threonine kinase mTOR, which is mainly regulated by PI3K/Akt signaling, is a crucial suppressor of autophagy (Datta et al., 2014). A recent study showed that the PI3K/AKT/mTOR signaling pathway is closely related to the regulation of autophagy and intestinal mucosal barrier function (Tanaka et al., 2008; Chen et al., 2017).

There is currently no effective therapeutic or preventative strategy for small bowel injury caused by NSAIDs (Utzeri and Usai, 2017). As a number of flavonoids participate in the regulation of intestinal TJ barrier integrity, they may be promising therapeutic agents. This regulation of intestinal TJ barrier integrity may partially contribute to the flavonoid-mediated biological effects on human health (Noda et al., 2012). Many natural products are rich in flavonoids and possess protective activities against intestinal inflammation, barrier integrity, and changes in gut microbiota (Gil-Cardoso et al., 2016). PTFC used in the present study were prepared from Qu Zhi Qiao (fruit of *Citrus Paradisi cv. Changshanhuyou*) which is one of the new "Zhejiang eight flavors." Qu Zhi Qiao was collected by "Standard for Processing Traditional Chinese Medicine in Zhejiang Province"which has the function of alleviating depression to regulate qi and relieving flatulence. In the current study, the PTFC contained narirutin, naringin, neohesperidin, and hesperidin, with a total flavonoid content of 76.22%. In recent years, underlying beneficial characteristics of PTFC and their metabolites have been investigated, including optimization of barrier permeability, positive balance of gut microbiota, and immunomodulation, as well as inhibition of oxidative stress and inflammation in gut, hepatoprotective and pro-cardiovascular activities (Vazhappilly et al., 2019; Shi et al., 2020; Wang et al., 2021). Evidence pointed to the health-promoting properties of hesperidin and narirutin, the most abundant citrus flavanones in citrus genus, on the intestinal barrier and human health (Shen et al., 2019; Guirro et al., 2020). In animal models with drug-induced intestinal epithelial damage, hesperidin manifested its protective effect by maintaining the intestinal epithelial barrier, most researchers stated the molecular basis for the effects seems to be mediated via decreased inflammatory mediators pathway (Tejada et al., 2018), but it may get involved in the autophagy pathways as well, though proposed in pathogenesis of diabetes via targeting TGF-β signaling presently (Heydarpour et al., 2020). Additionally, even hesperetin, the aglycones of hesperidin, has also been shown to ameliorate the epithelial barrier damage via increased expressions of TJ proteins ZO-1 and OCLN (Zhang et al., 2020), which is consistent with our findings. The similarly enhancement of TJ integrity *in vivo* and *in vitro*, induced by citrus-derived flavonoids, naringin, was also demonstrated. That exerted significant effects on alleviating sepsis-induced intestinal mucosal injury and improving impaired intestinal permeability (Li et al., 2018). Our previous study even directly proved its protective effect on small intestine injury in NSAIDs related enteropathy (Chao et al., 2021). More importantly, emerging research reported that naringin attenuated the severity of colitis by suppressing endoplasmic reticulum stress-induced autophagy in colorectal mucosal cells (Pei et al., 2020), and regulated autophagy mediated by PI3K-Akt-mTOR pathway to ameliorate endothelial cell dysfunction induced by high glucose/high fat stress (Wang et al., 2020), or decrease hepatic stellate cell activity in liver fibrosis (Shi et al., 2016), the same as the autophagy regulatory pathway of NSAIDs intestinal disease in this study. While another component, neohesperidin, also derived from flavonoids, though a study indicatied that it might not affect the TJ (Nakashima et al., 2020), it offered virtually complete protection against the autophagy-inhibitory effect of okadaic acid. Therefore, these four compounds may play key roles in regulating autophagy and the integrity of the intestinal TJ barrier. First, our data showed that expression levels of Atg5 and LC3-II and the ratio of LC3-II/LC3-I decreased in the NSAID model group compared to those in the control group, whereas the PTFC group exhibited opposite results. Second, our results showed that the expression levels of p-PI3K, p-Akt, p-mTOR, and p-PI3K/PI3K as well as the ratios of p-Akt/Akt and p-mTOR/mTOR were higher in the NSAID model group and lower in the PTFC group compared to those in the control group. These findings suggest that PTFC activated autophagy and inhibited the progression of NSAID-induced small intestine disease, which was mediated, at least partly, by the PI3K/AKT signaling pathway.

## Conclusion

Our data presents new information regarding increased autophagy levels, which protected rats against NSAID-induced small intestine disease. In addition, the PI3K/AKT pathway was identified to be functionally vital for attenuating autophagy. Our results also indicate that pretreatment with PTFC attenuated NSAID-induced small intestine injury and protected the intestinal mucosal barrier of rats. These findings have the potential for translation into novel protective strategies. Finally, PTFC may be considered a therapeutic candidate for NSAID-induced small intestine injury. Future clinical trials will be needed to confirm the effective human dose of PTFC.

## Data Availability

The raw data supporting the conclusions of this article will be made available by the authors, without undue reservation, to any qualified researcher.
