# Supplementary figures and images for: Pure Total Flavonoids From Citrus Protect Against Nonsteroidal Anti-inflammatory Drug-Induced Small Intestine Injury by Promoting Autophagy in vivo and in vitro
Source: Front Pharmacol. 2021 Apr 19;12:622744. doi: 10.3389/fphar.2021.622744 (PMC8090934; doi:10.3389/fphar.2021.622744)

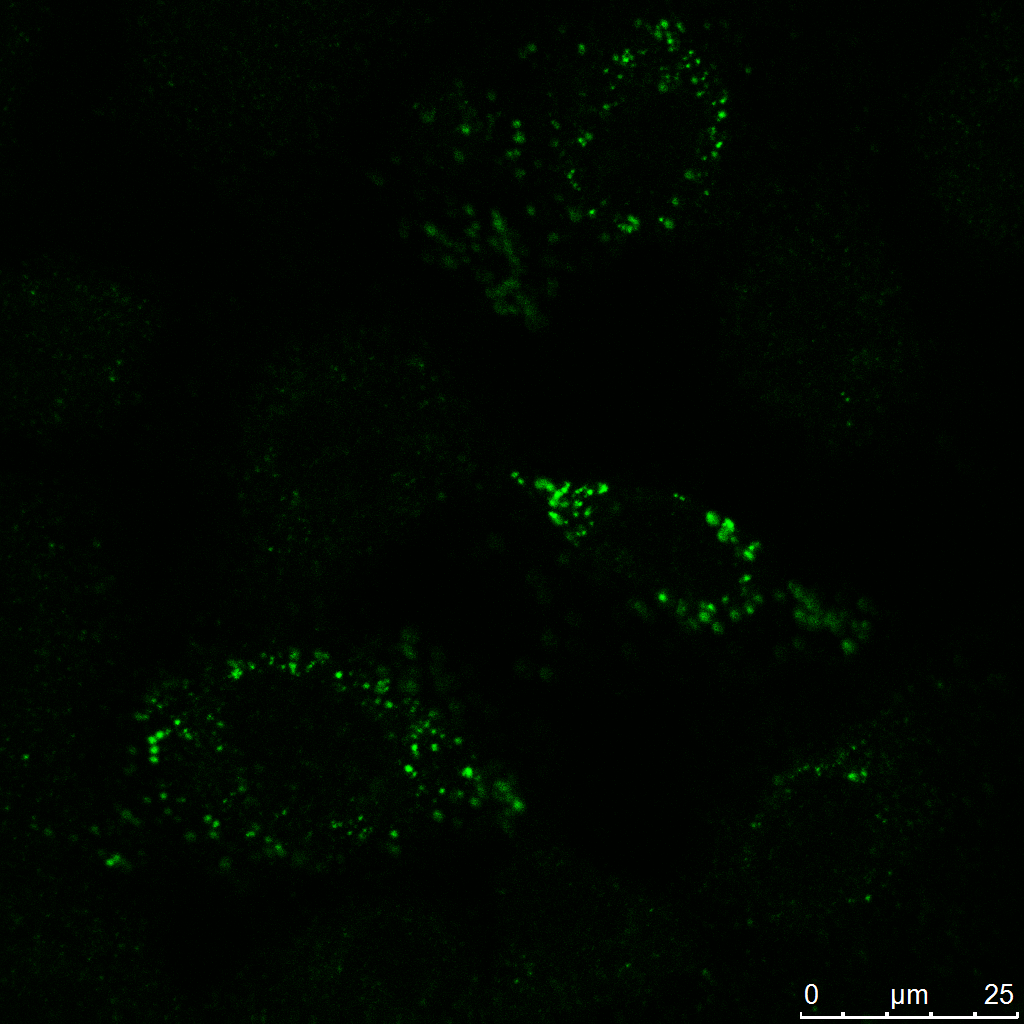

Supplement: Supplementary file 1 [file datasheet1.zip › PTFC Figures-Immunofluorescence staining/PTFC-Green.tif]

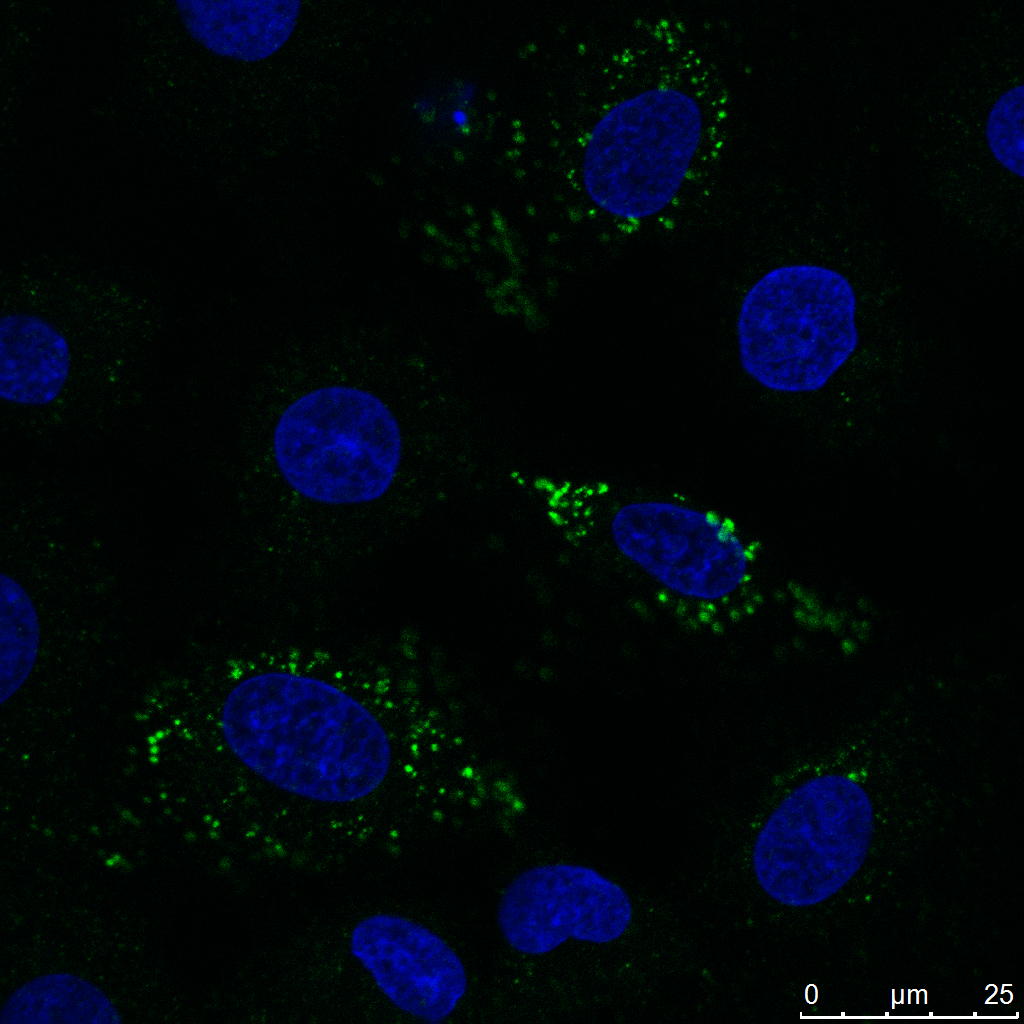

Supplement: Supplementary file 1 [file datasheet1.zip › PTFC Figures-Immunofluorescence staining/PTFC-Merge.tif]

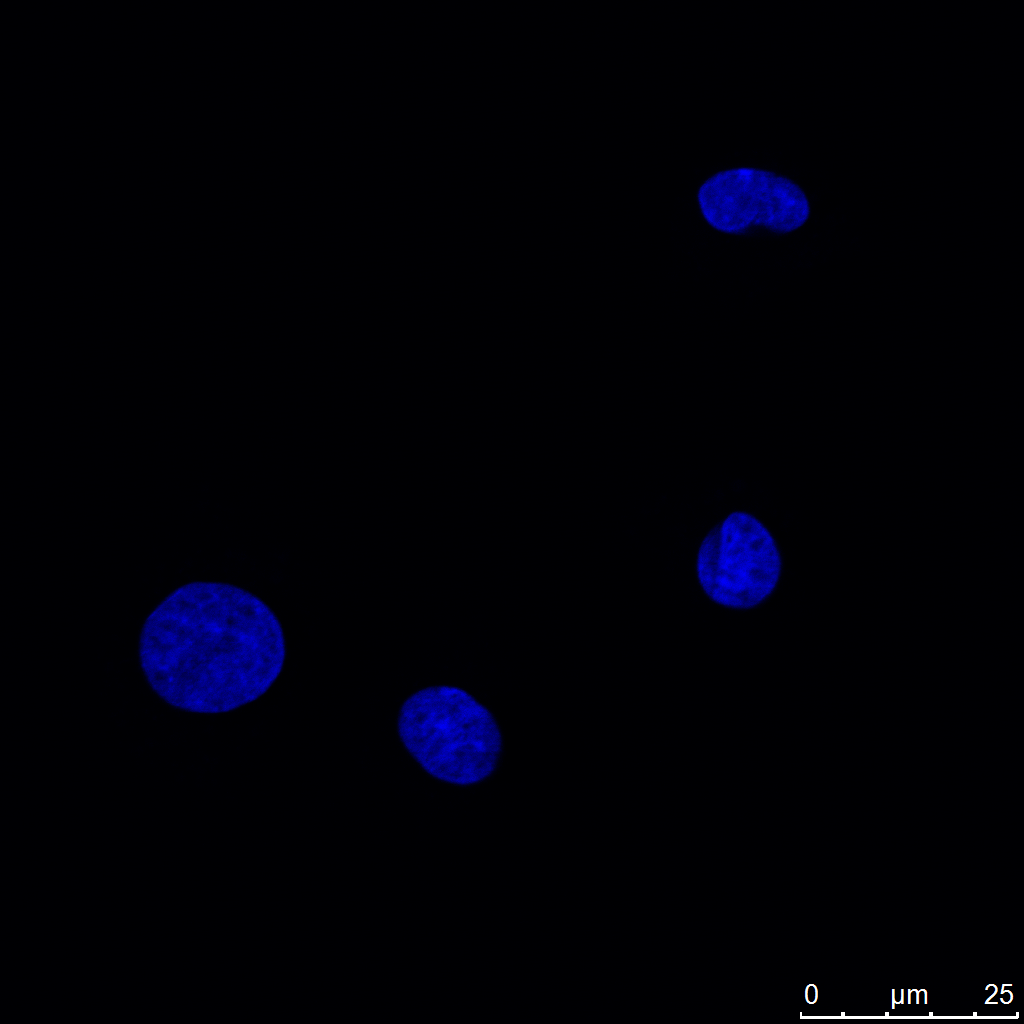

Supplement: Supplementary file 1 [file datasheet1.zip › PTFC Figures-Immunofluorescence staining/50 ╬╝M Chloroquine-DAPI.tif]

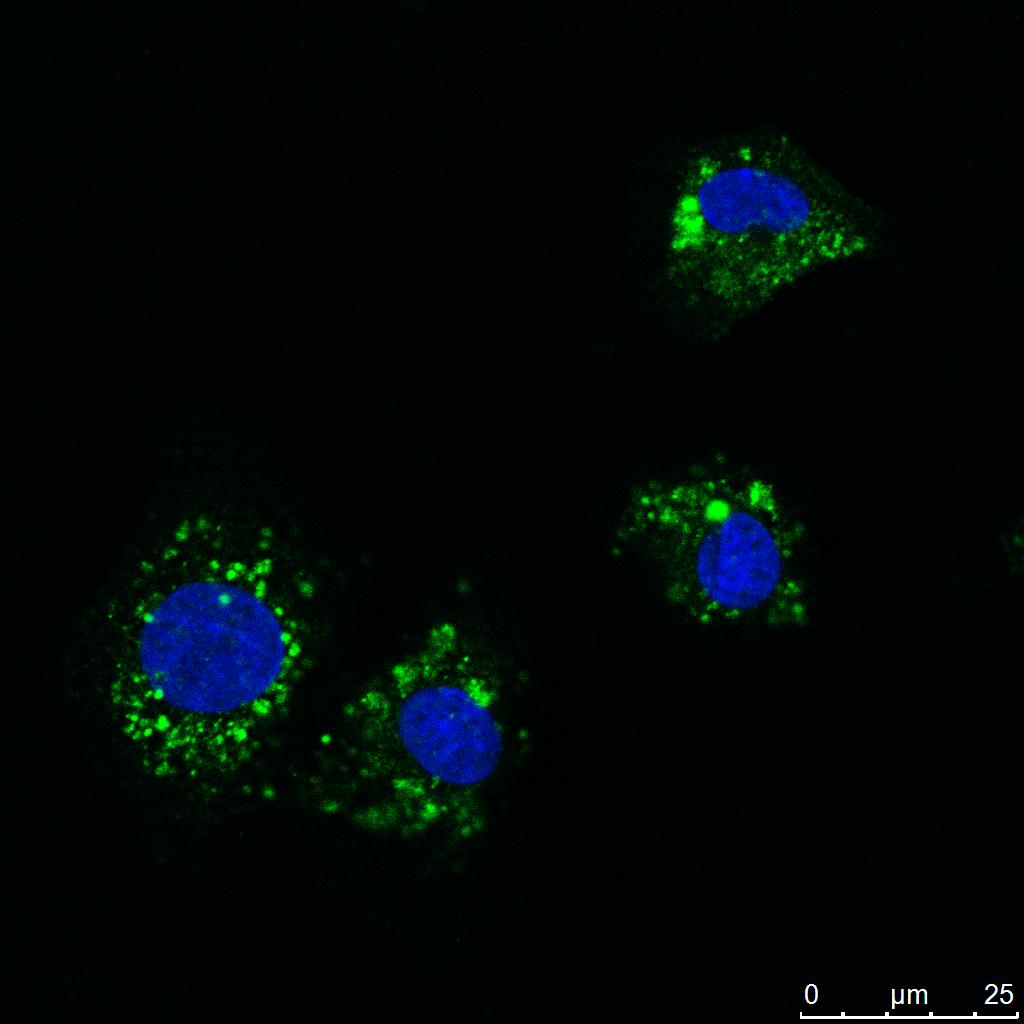

Supplement: Supplementary file 1 [file datasheet1.zip › PTFC Figures-Immunofluorescence staining/50 ╬╝M Chloroquine-Merge.tif]

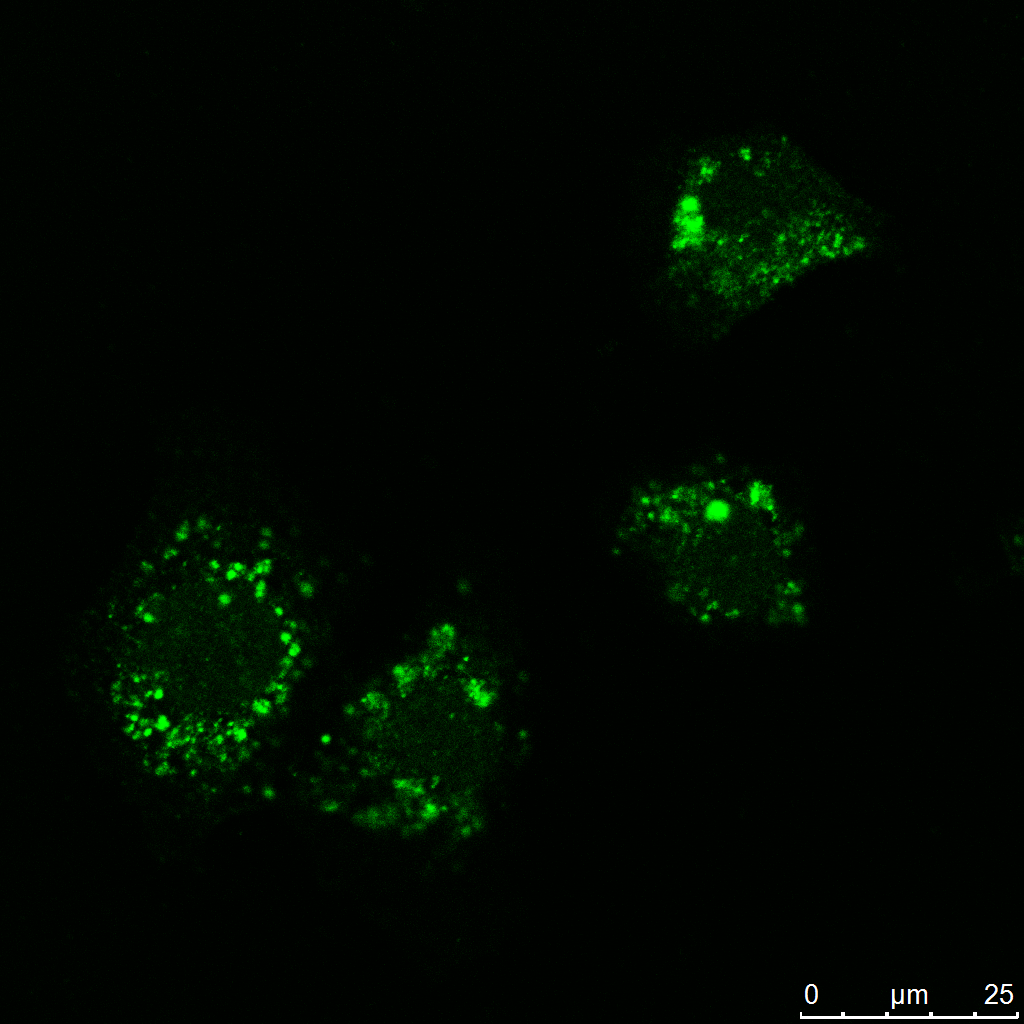

Supplement: Supplementary file 1 [file datasheet1.zip › PTFC Figures-Immunofluorescence staining/50 ╬╝M Chloroquine-Green.tif]

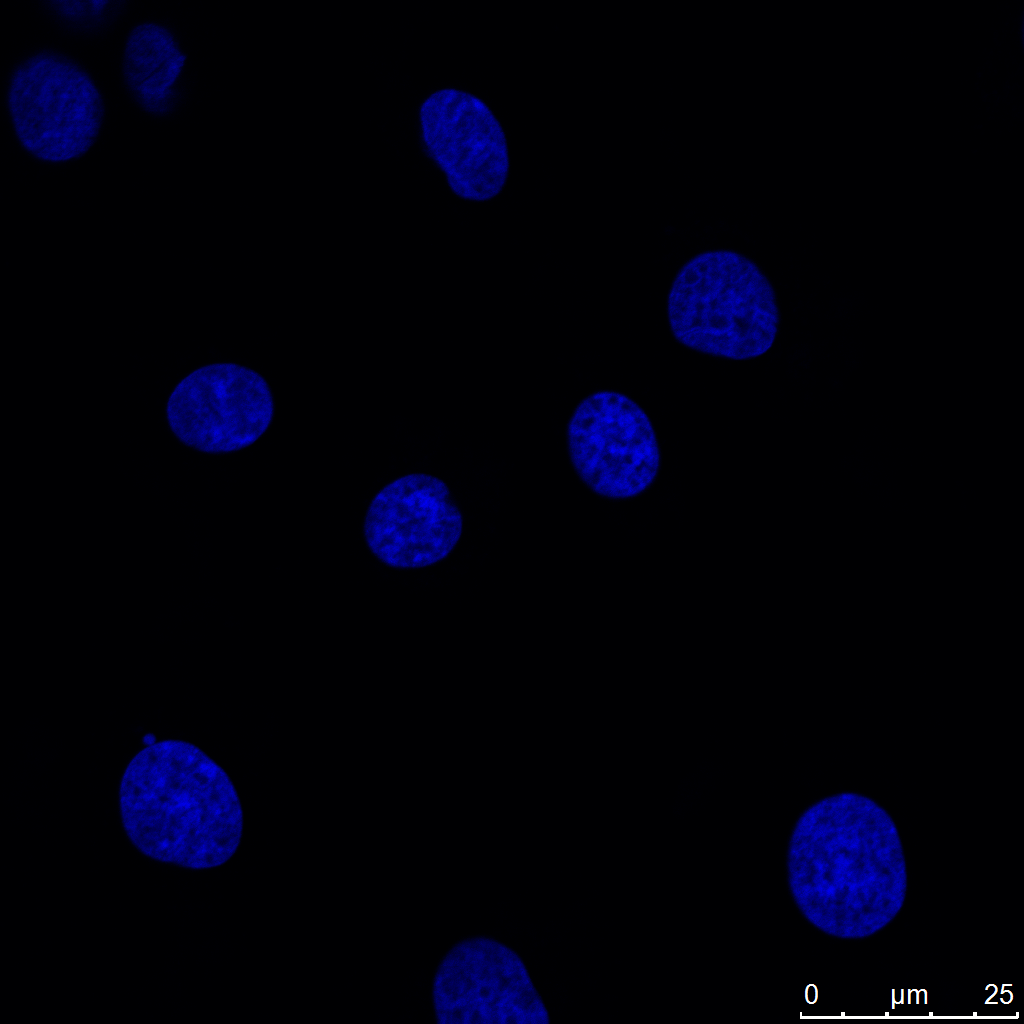

Supplement: Supplementary file 1 [file datasheet1.zip › PTFC Figures-Immunofluorescence staining/Control-DAPI.tif]

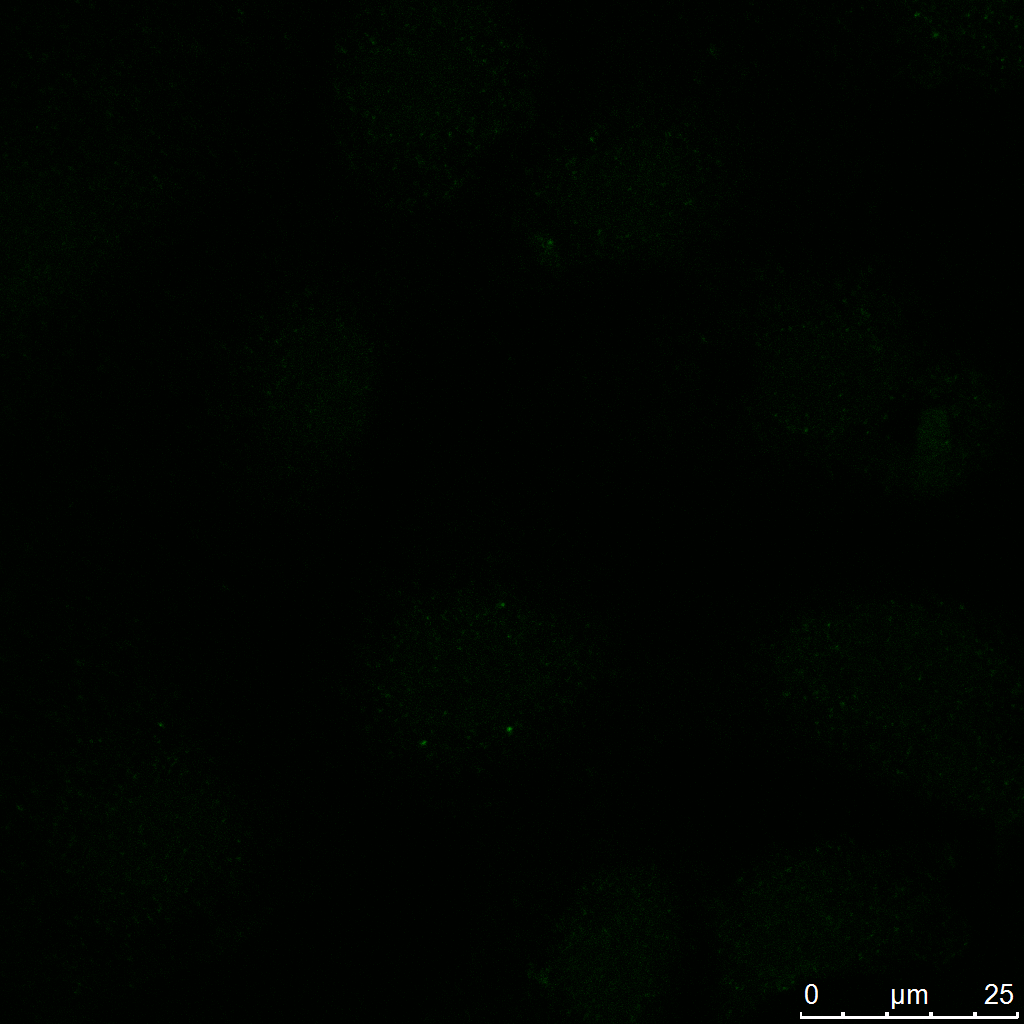

Supplement: Supplementary file 1 [file datasheet1.zip › PTFC Figures-Immunofluorescence staining/NSAIDs-Green.tif]

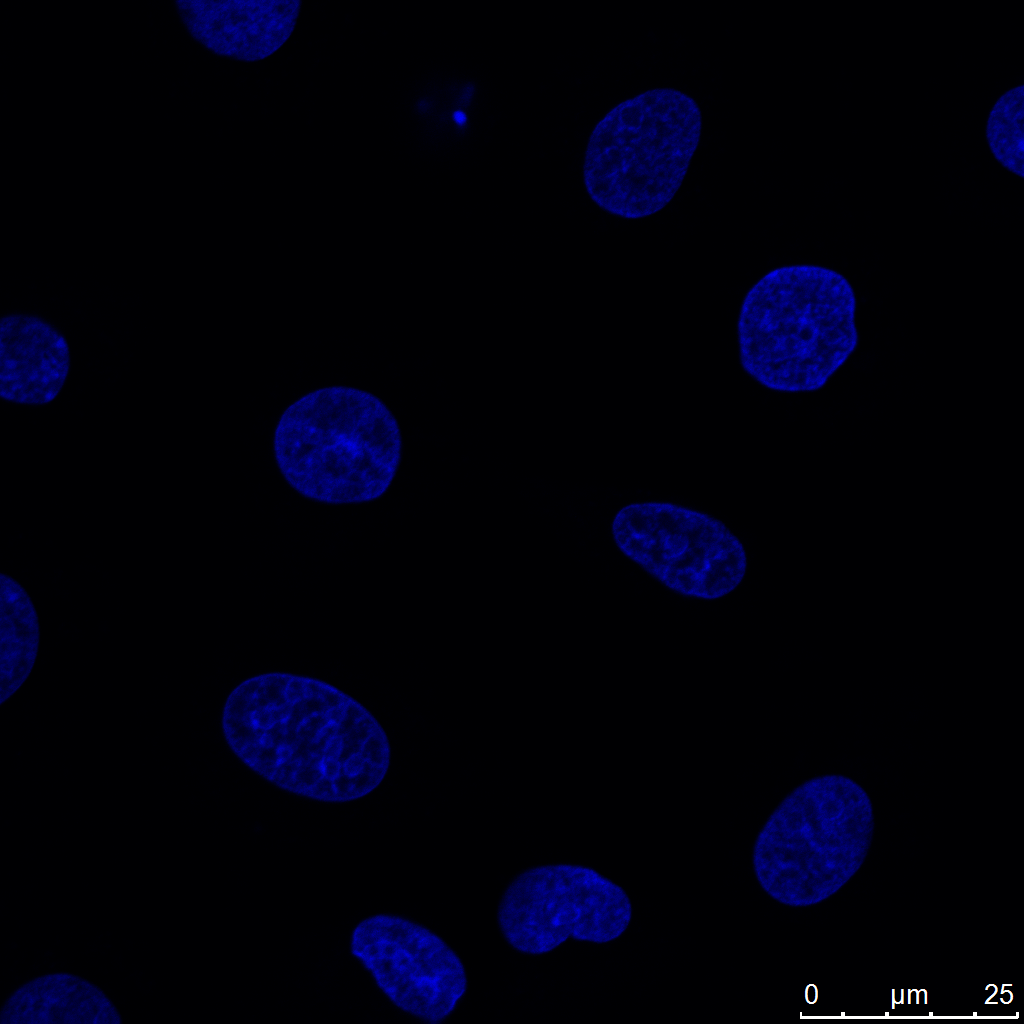

Supplement: Supplementary file 1 [file datasheet1.zip › PTFC Figures-Immunofluorescence staining/PTFC-DAPI.tif]

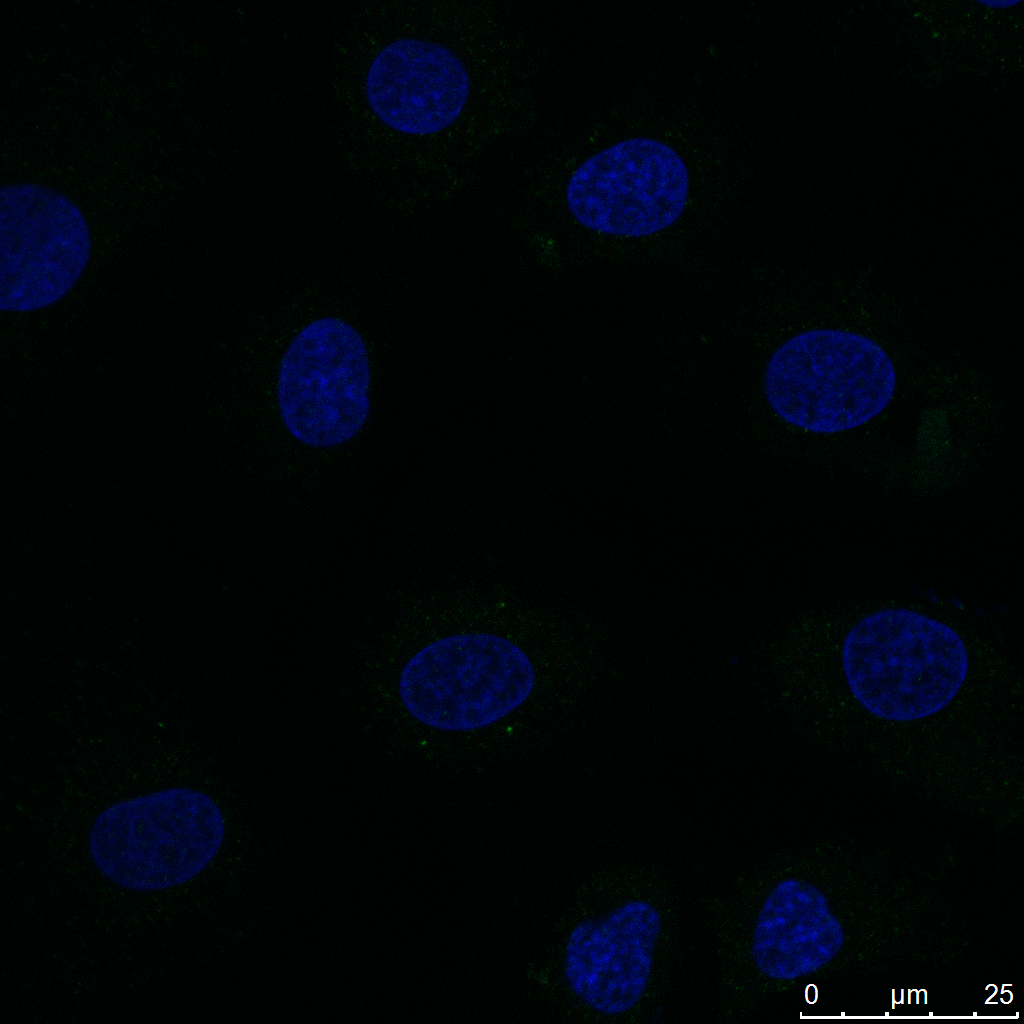

Supplement: Supplementary file 1 [file datasheet1.zip › PTFC Figures-Immunofluorescence staining/NSAIDs-Merge.tif]

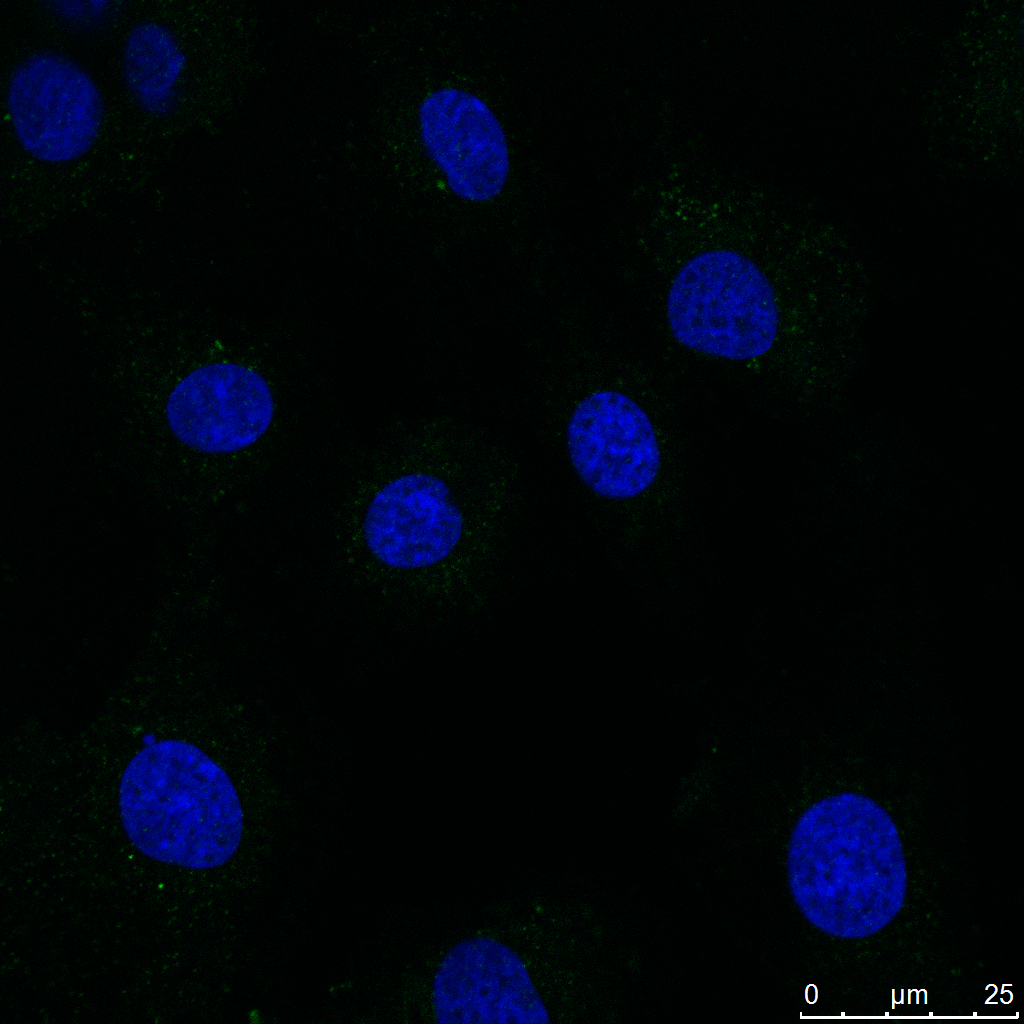

Supplement: Supplementary file 1 [file datasheet1.zip › PTFC Figures-Immunofluorescence staining/Control-Merge.tif]

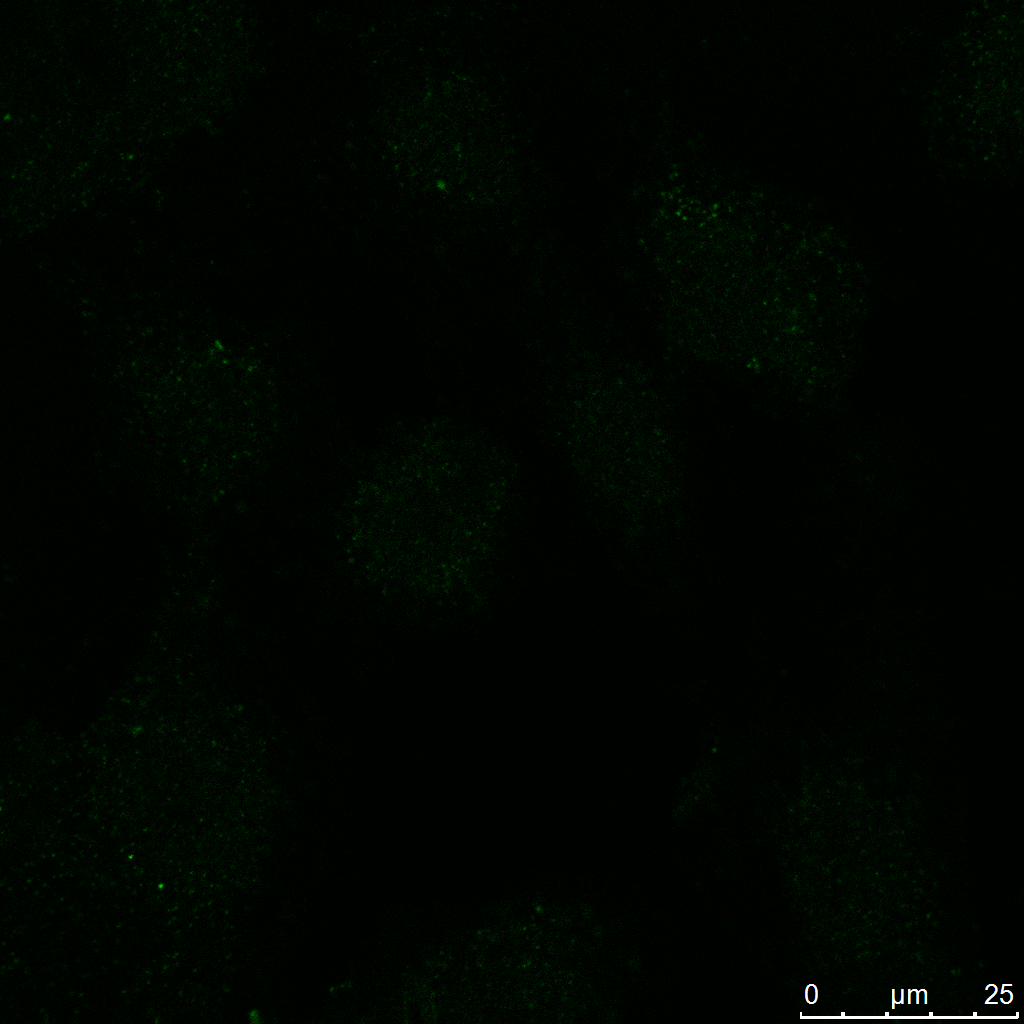

Supplement: Supplementary file 1 [file datasheet1.zip › PTFC Figures-Immunofluorescence staining/Control-Green.tif]

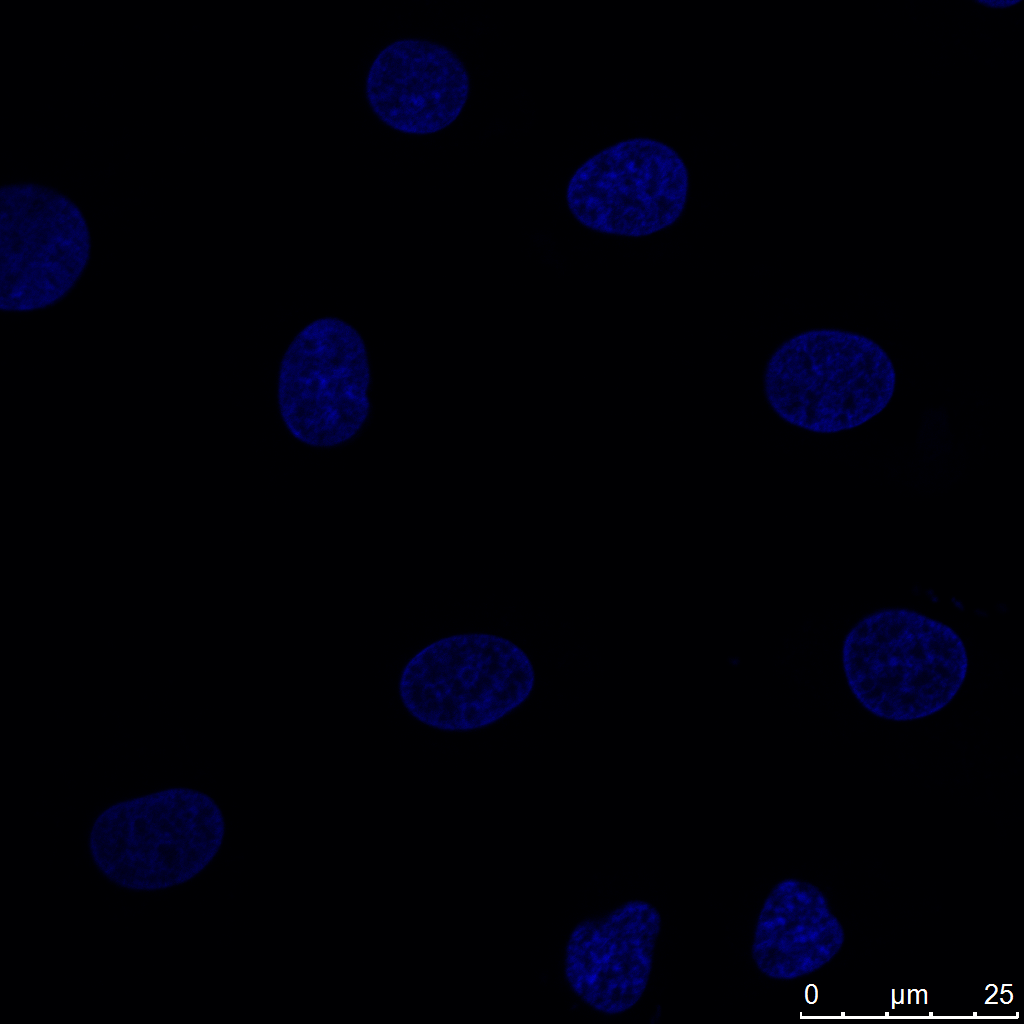

Supplement: Supplementary file 1 [file datasheet1.zip › PTFC Figures-Immunofluorescence staining/NSAIDs-DAPI.tif]

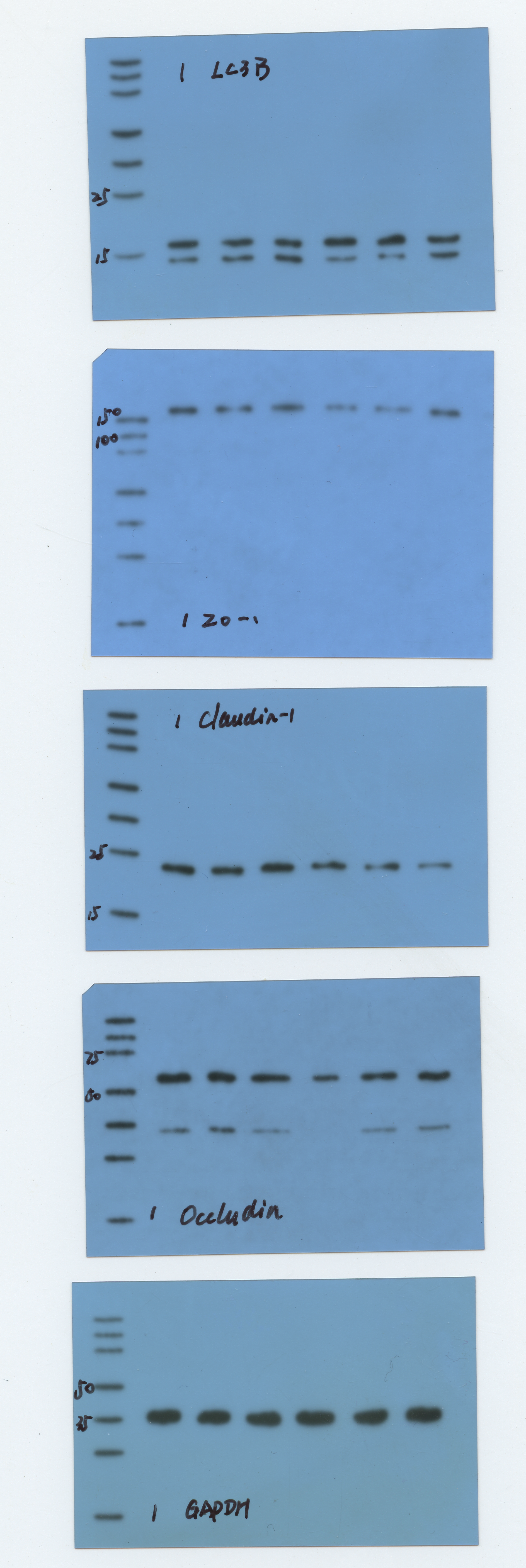

Supplement: Supplementary file 2 [file datasheet2.zip › PTFC WB-1∩╝êfigure2∩╝ë/WB-1.tif]

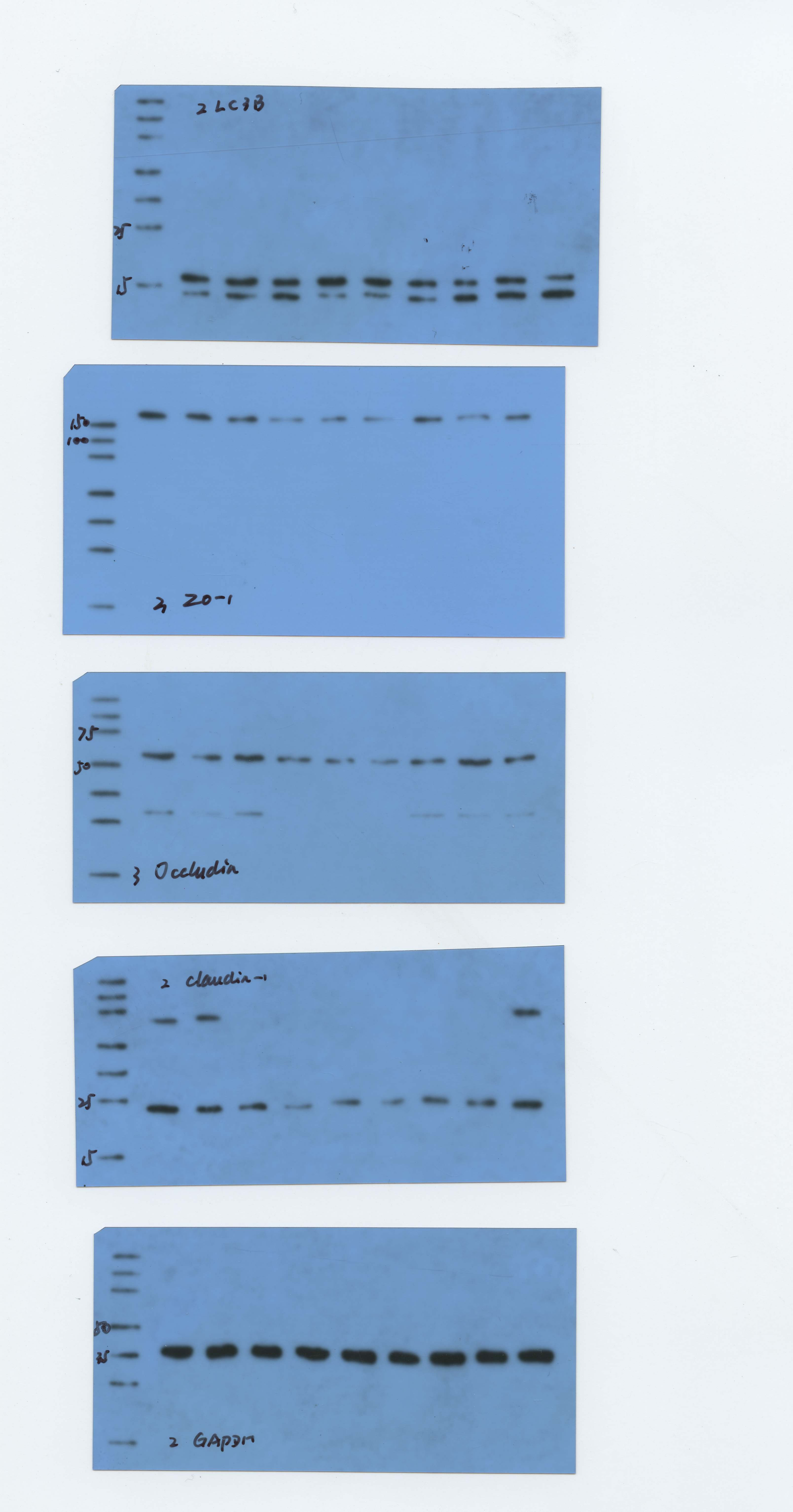

Supplement: Supplementary file 3 [file datasheet3.zip › PTFC WB-2∩╝êfigure4∩╝ë /WB-2.tif]

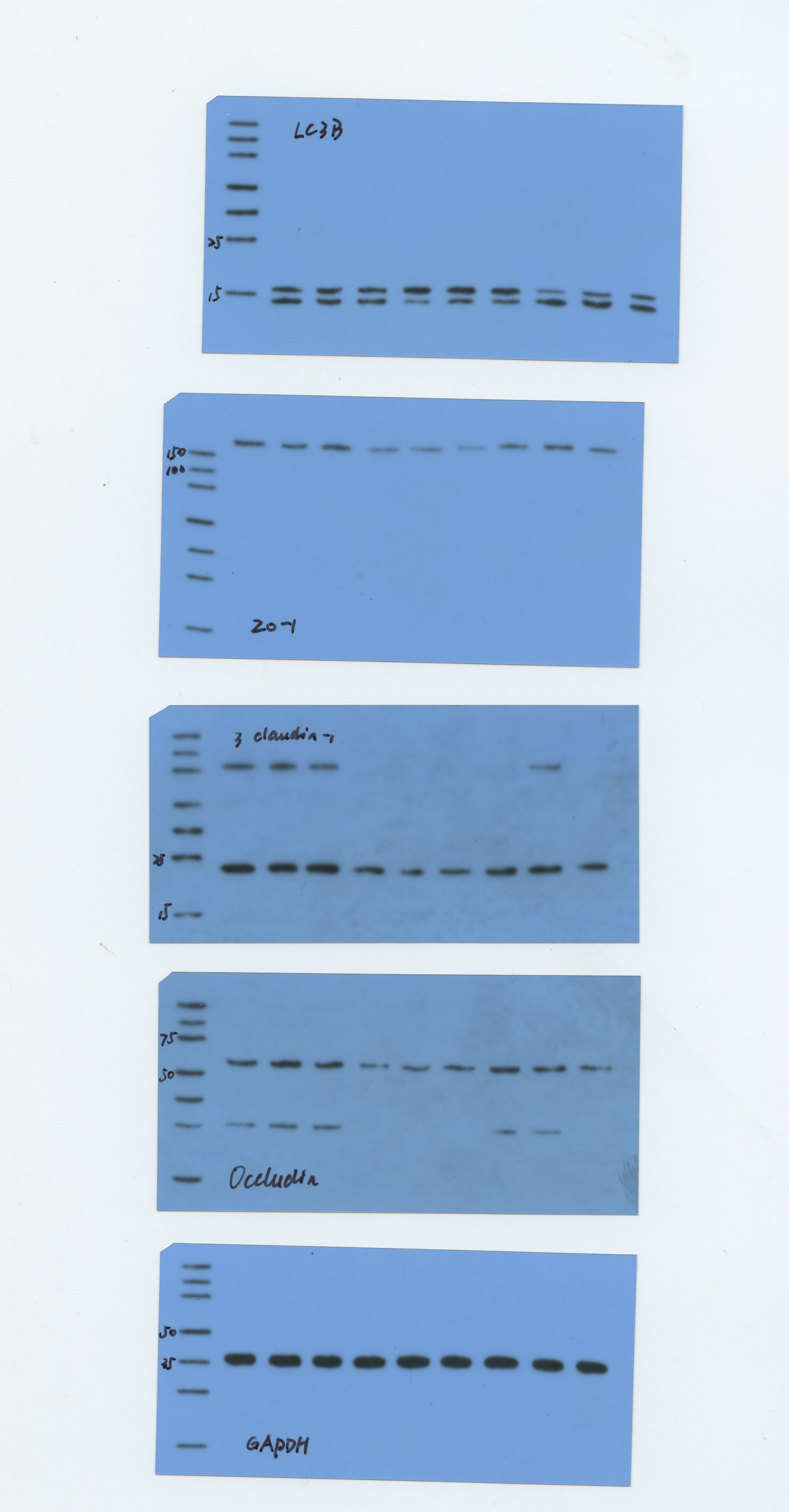

Supplement: Supplementary file 4 [file datasheet4.zip › PTFC WB-3∩╝êfigure5∩╝ë /WB-3.tif]

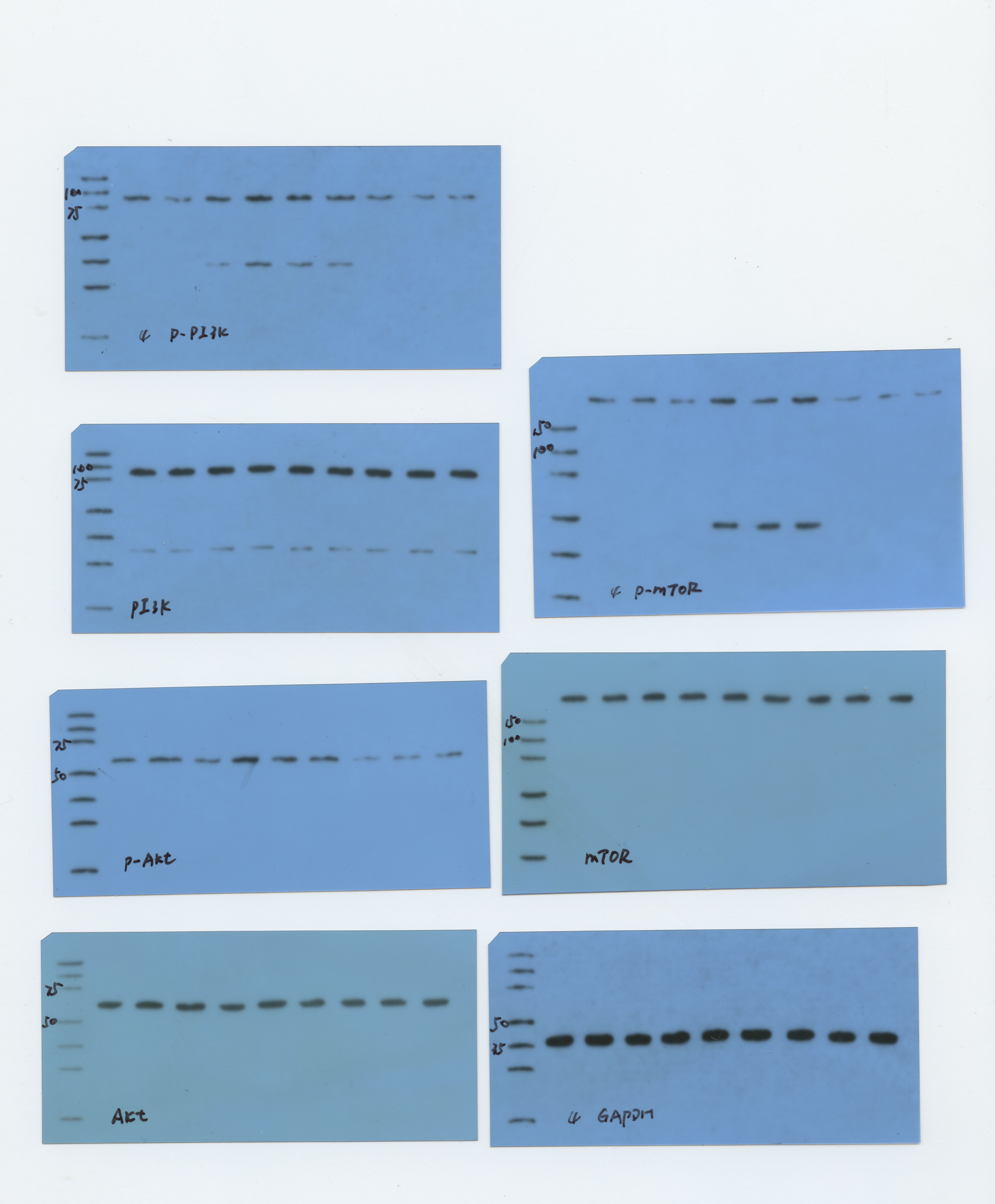

Supplement: Supplementary file 5 [file datasheet5.zip › PTFC WB-4 ∩╝êfigure7∩╝ë/WB-4.tif]

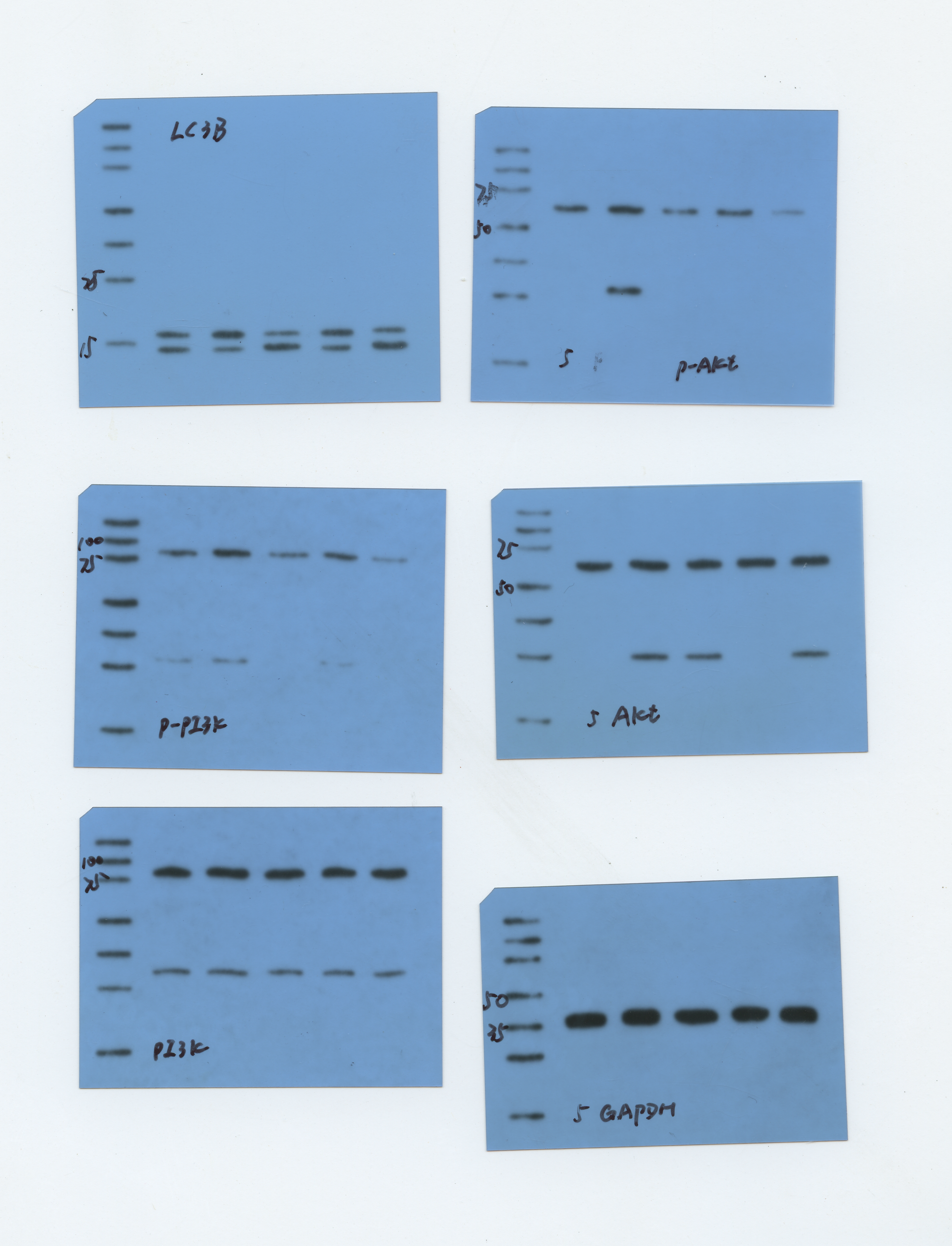

Supplement: Supplementary file 6 [file datasheet6.zip › PTFC WB-5∩╝êfigure8∩╝ë/WB-5.tif]

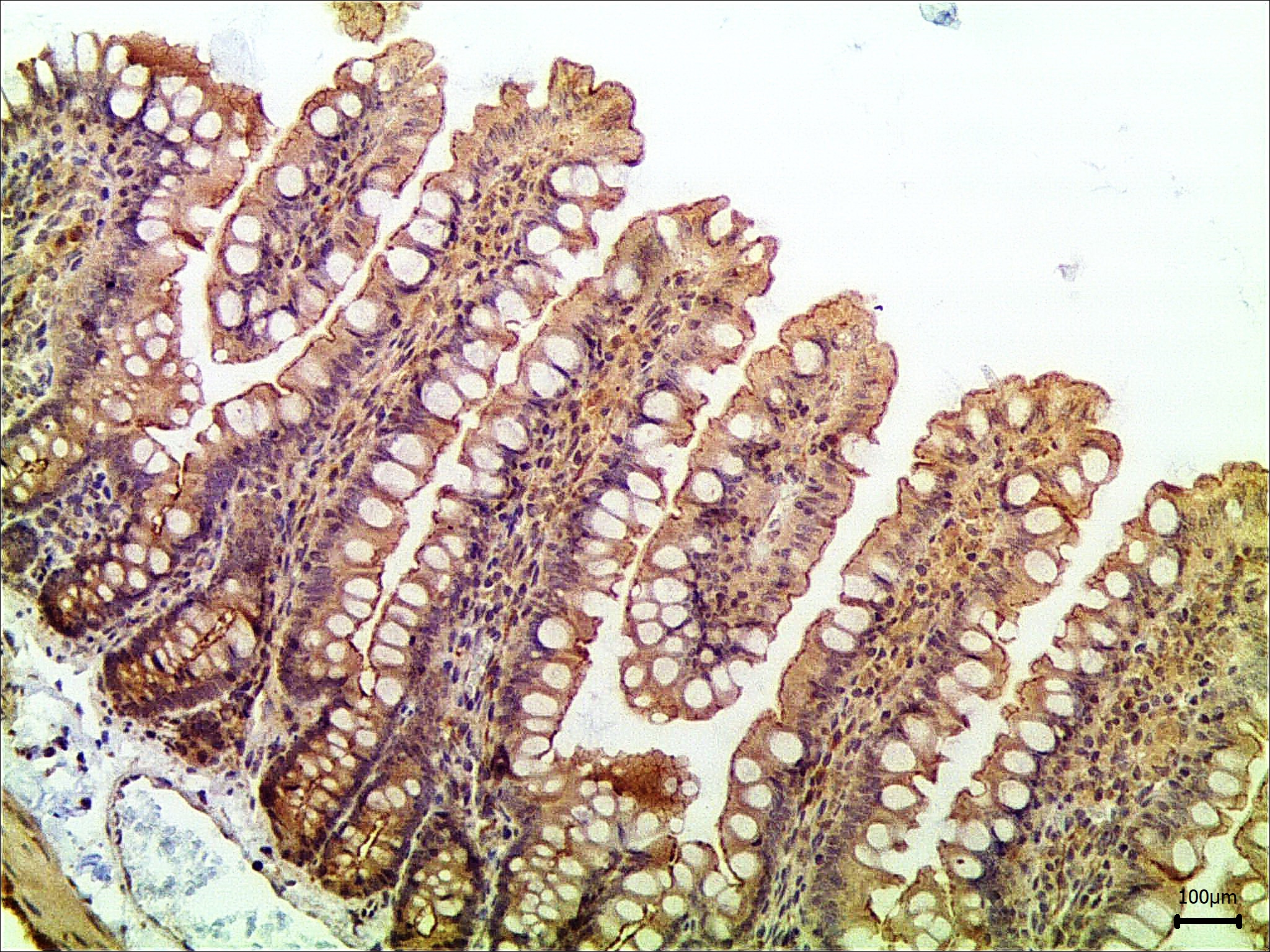

Supplement: Supplementary file 7 [file datasheet7.zip › PTFC Figures-IHC1/LC3B-PTFC.jpg]

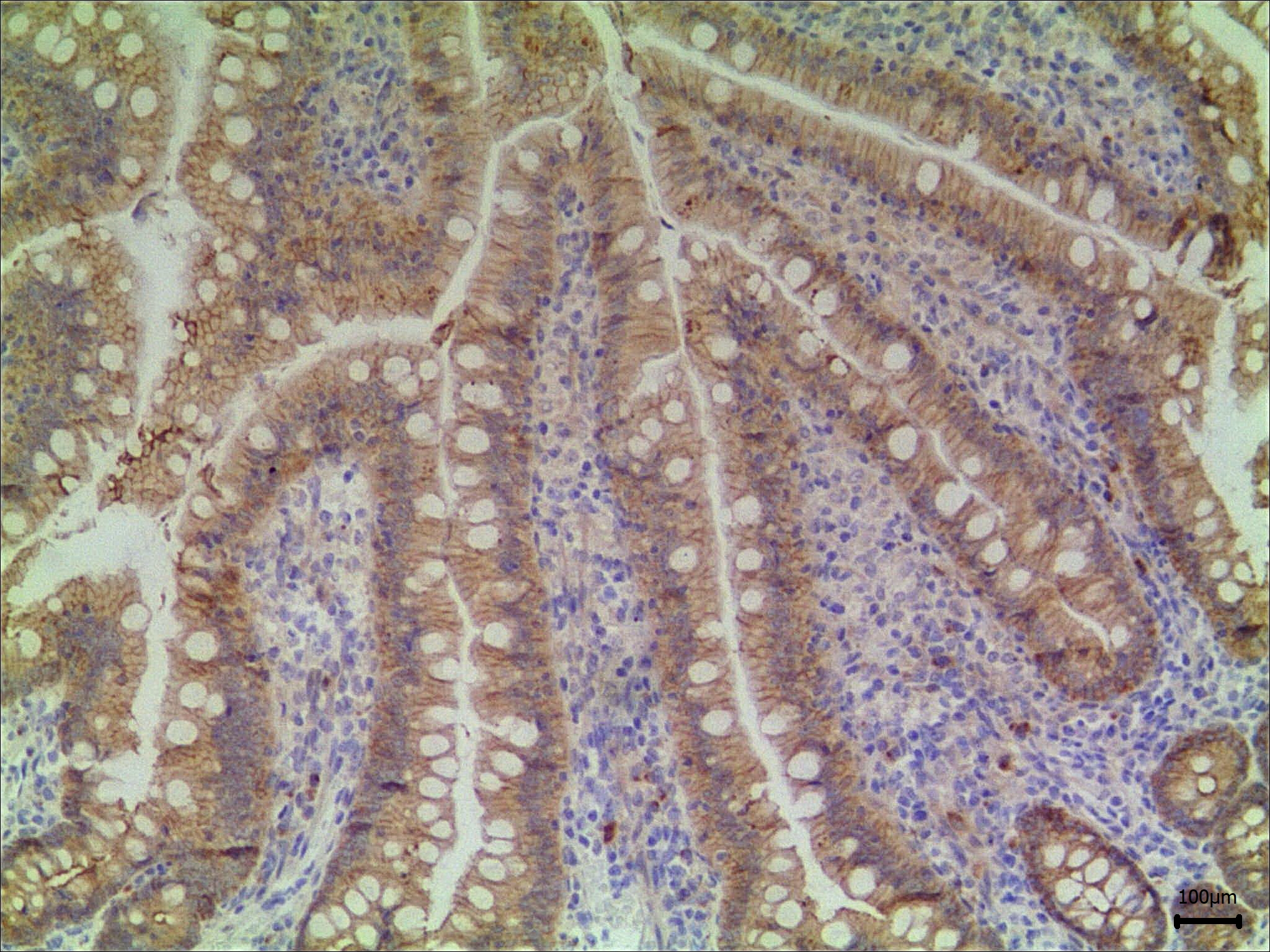

Supplement: Supplementary file 7 [file datasheet7.zip › PTFC Figures-IHC1/claudin-1-PTFC.jpg]

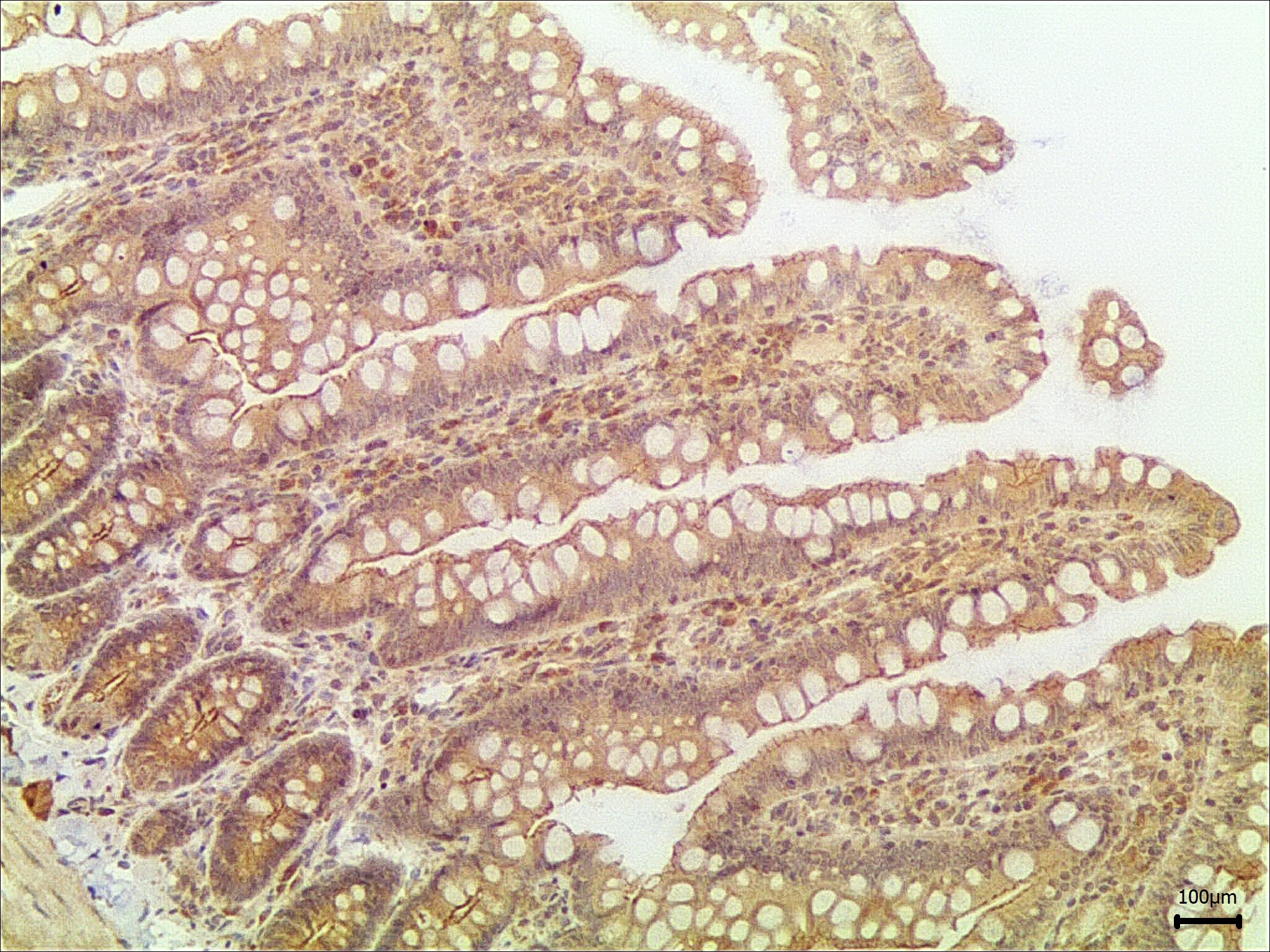

Supplement: Supplementary file 7 [file datasheet7.zip › PTFC Figures-IHC1/LC3B-control.jpg]

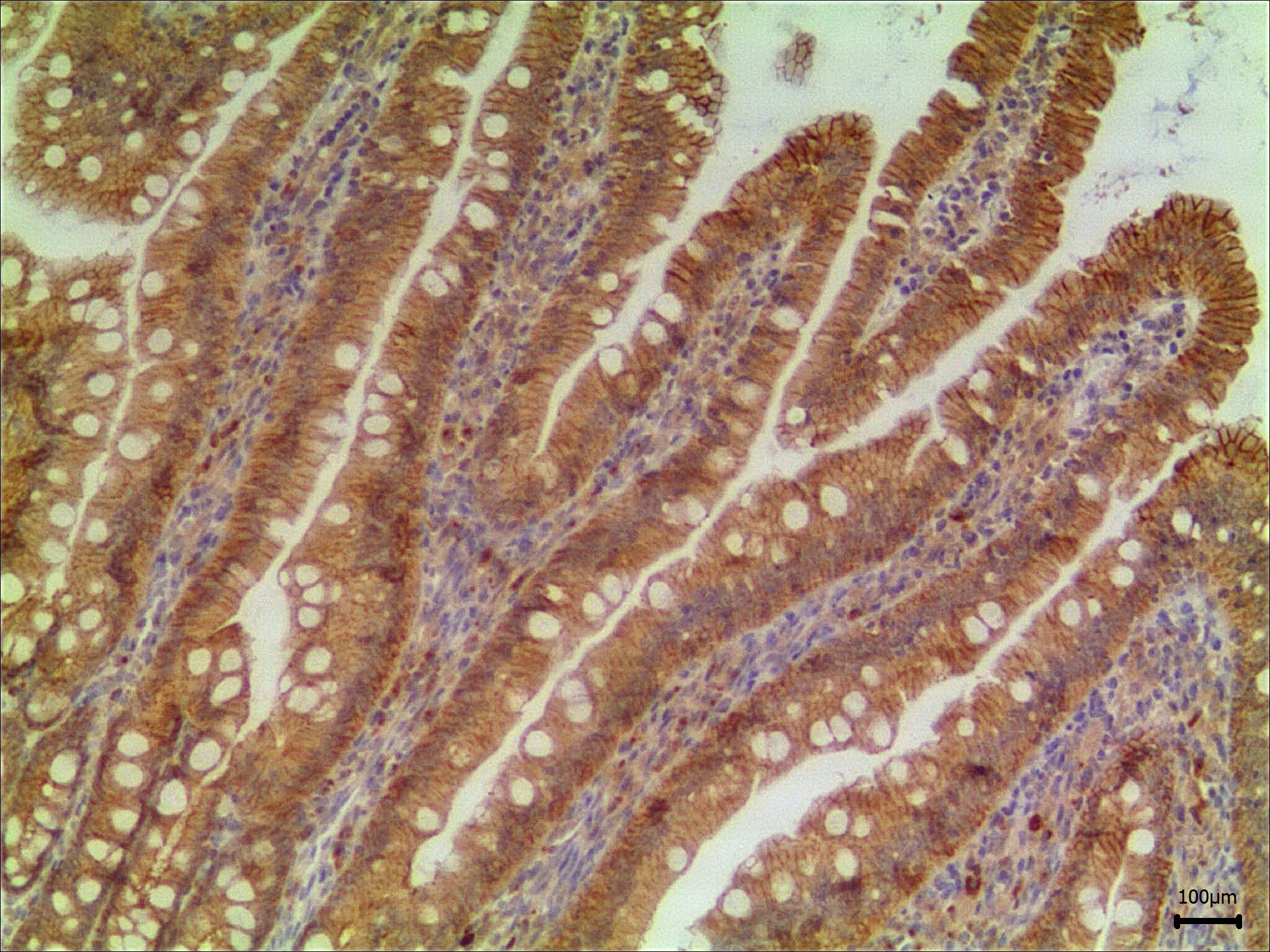

Supplement: Supplementary file 7 [file datasheet7.zip › PTFC Figures-IHC1/claudin-1-control.jpg]

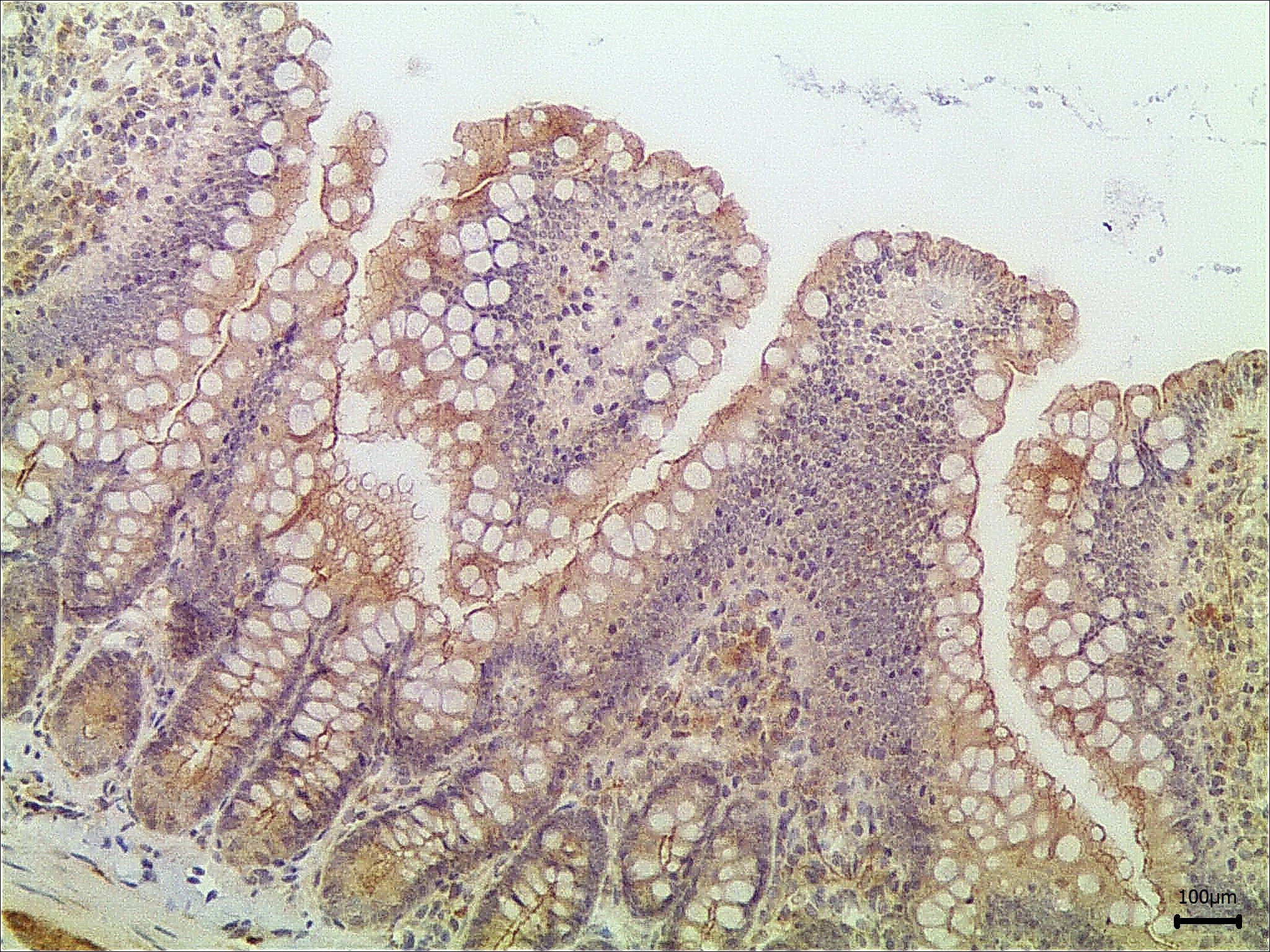

Supplement: Supplementary file 7 [file datasheet7.zip › PTFC Figures-IHC1/LC3B-NSAIDs.jpg]

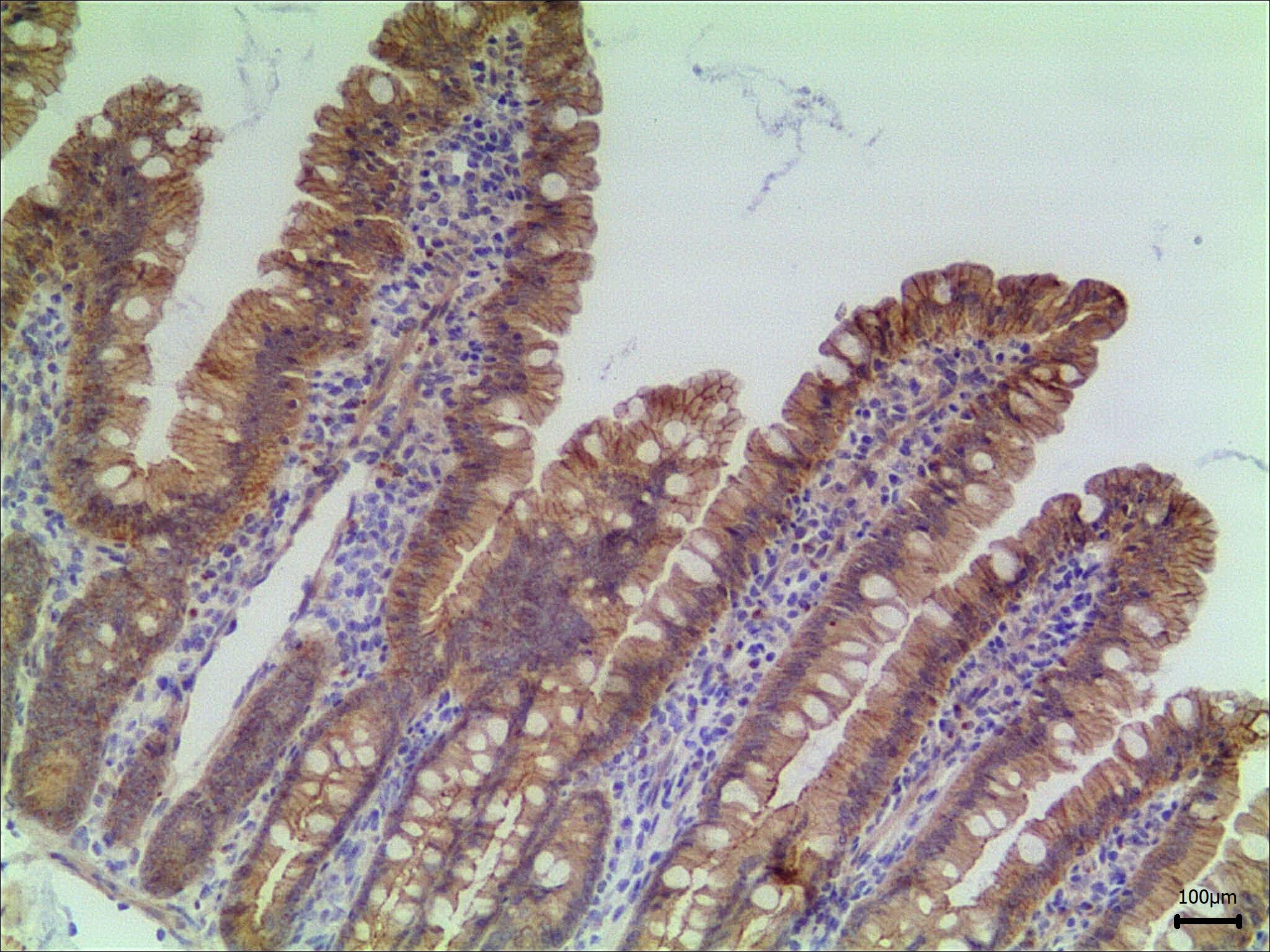

Supplement: Supplementary file 7 [file datasheet7.zip › PTFC Figures-IHC1/claudin-1-NSAIDs.jpg]

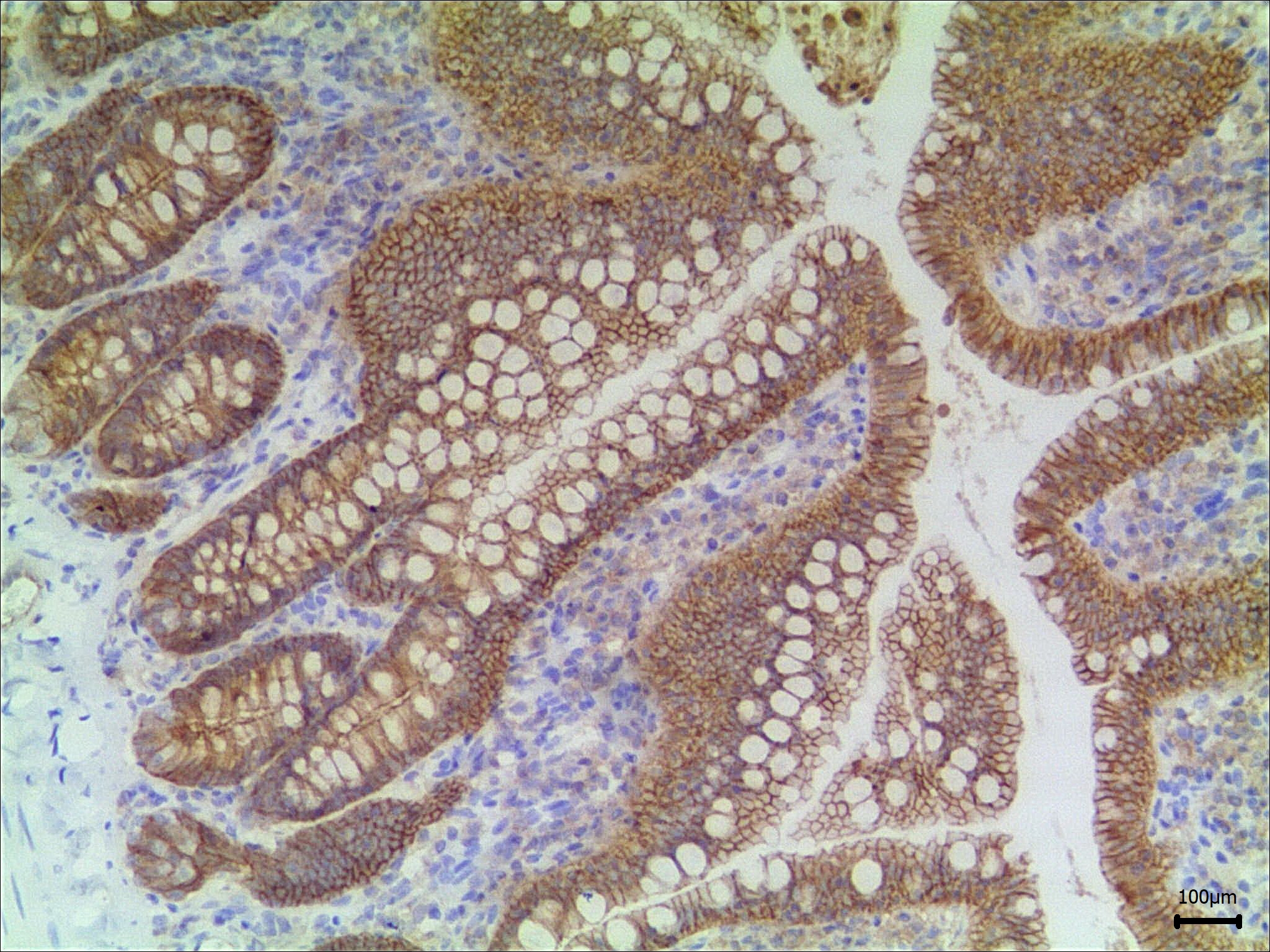

Supplement: Supplementary file 8 [file datasheet8.zip › PTFC Figures-IHC2/occludin-PTFC.jpg]

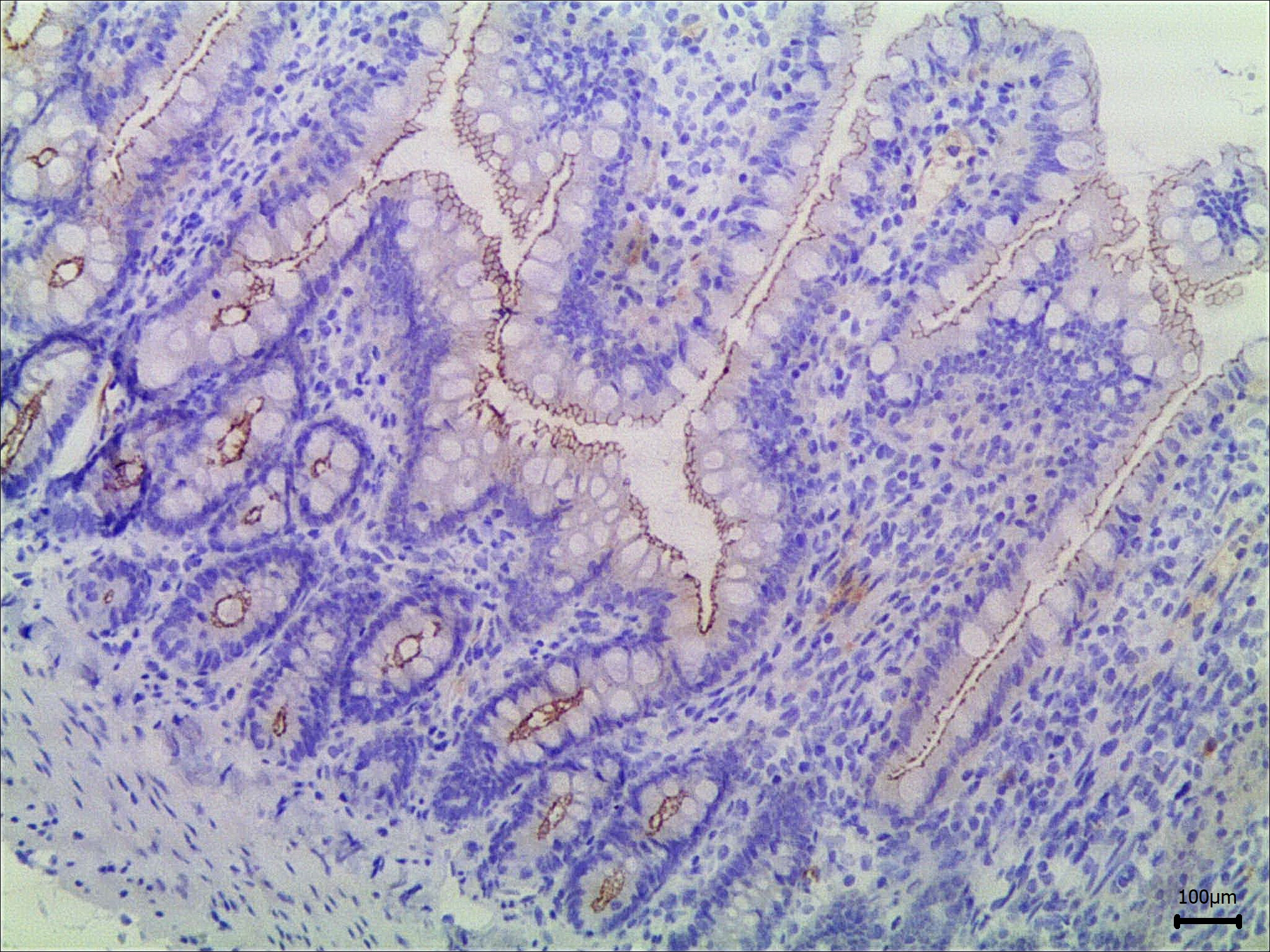

Supplement: Supplementary file 8 [file datasheet8.zip › PTFC Figures-IHC2/zo-1-NSAIDs.jpg]

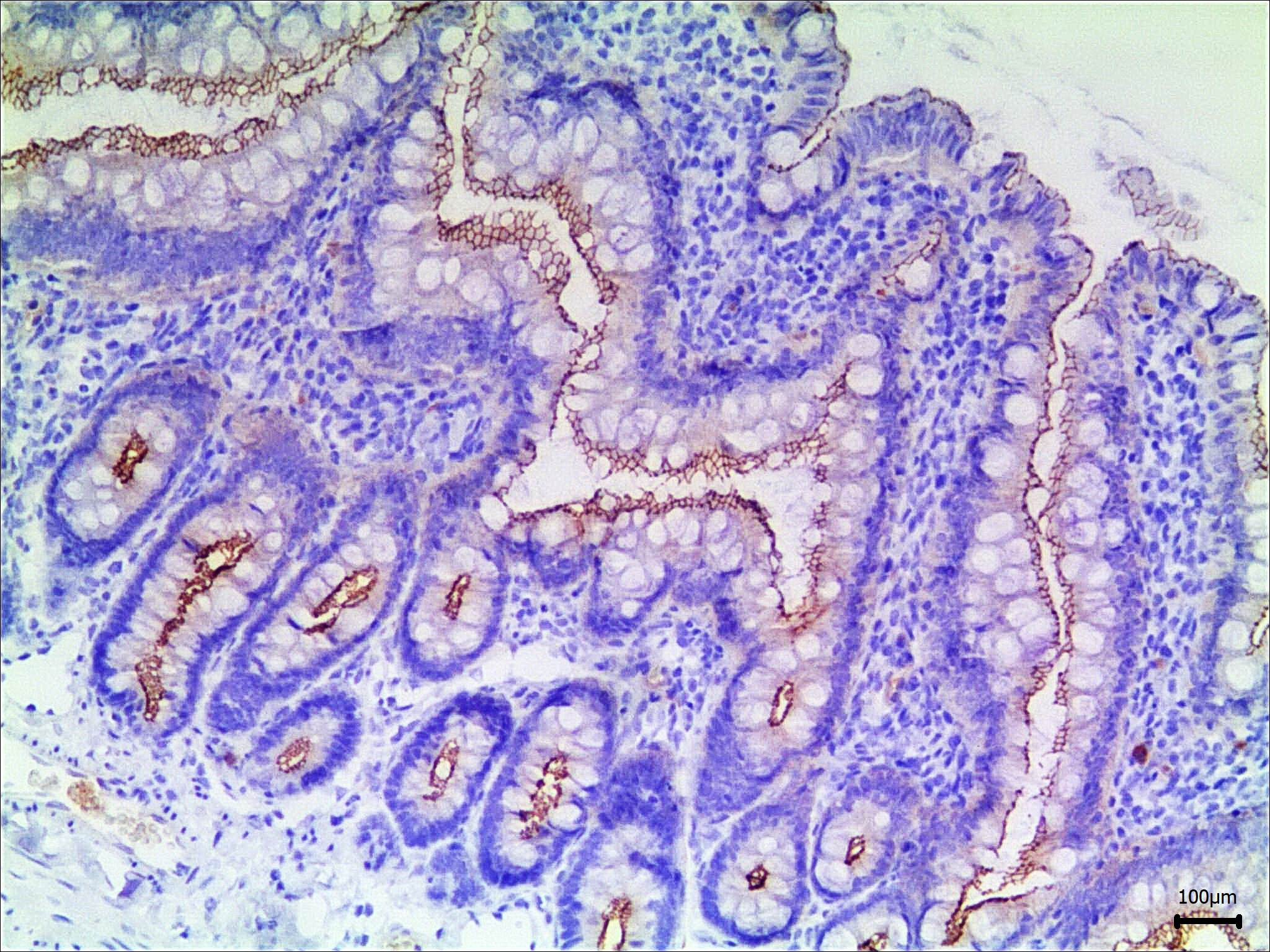

Supplement: Supplementary file 8 [file datasheet8.zip › PTFC Figures-IHC2/zo-1-control.jpg]

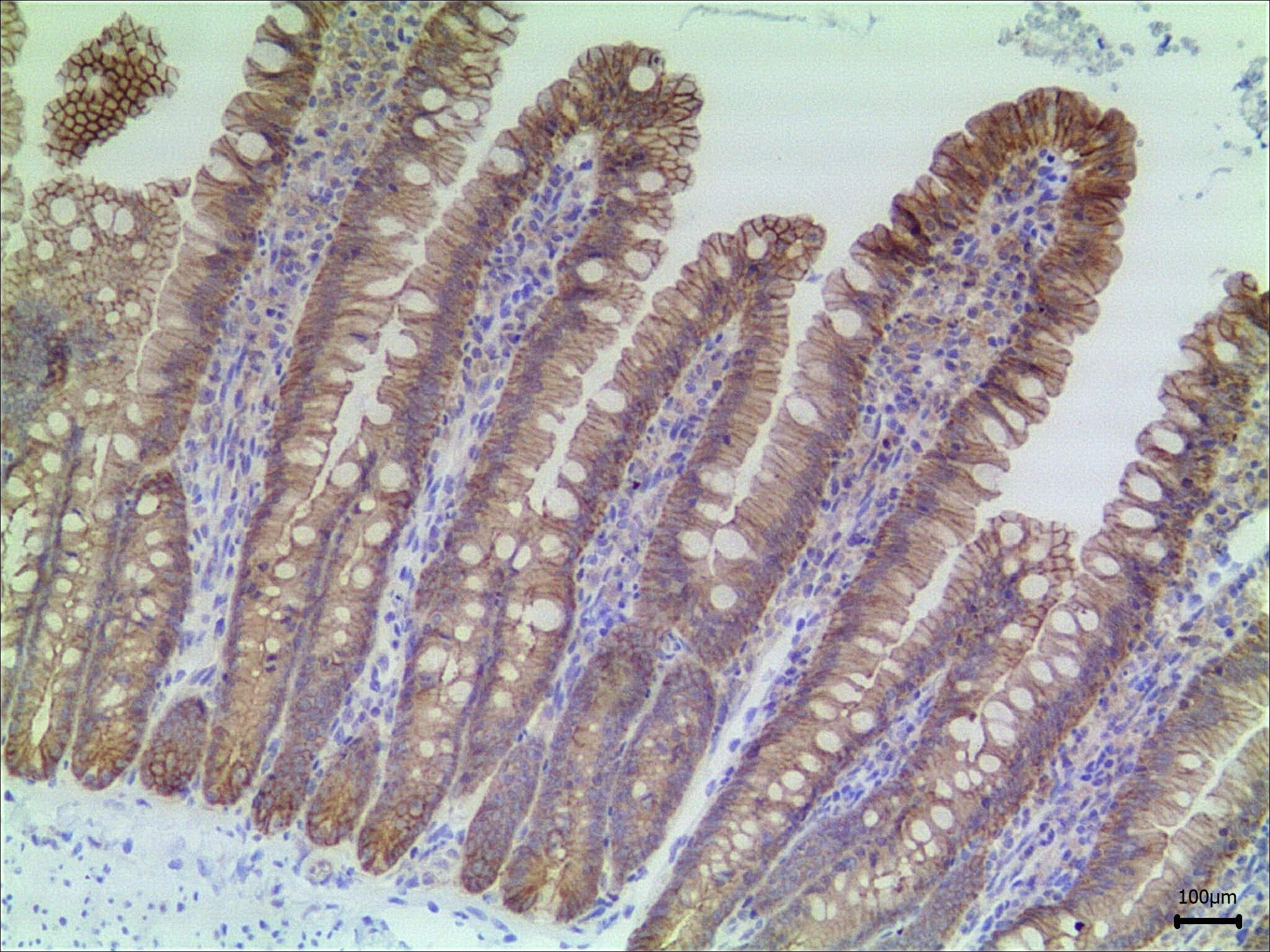

Supplement: Supplementary file 8 [file datasheet8.zip › PTFC Figures-IHC2/occludin-NSAIDs.jpg]

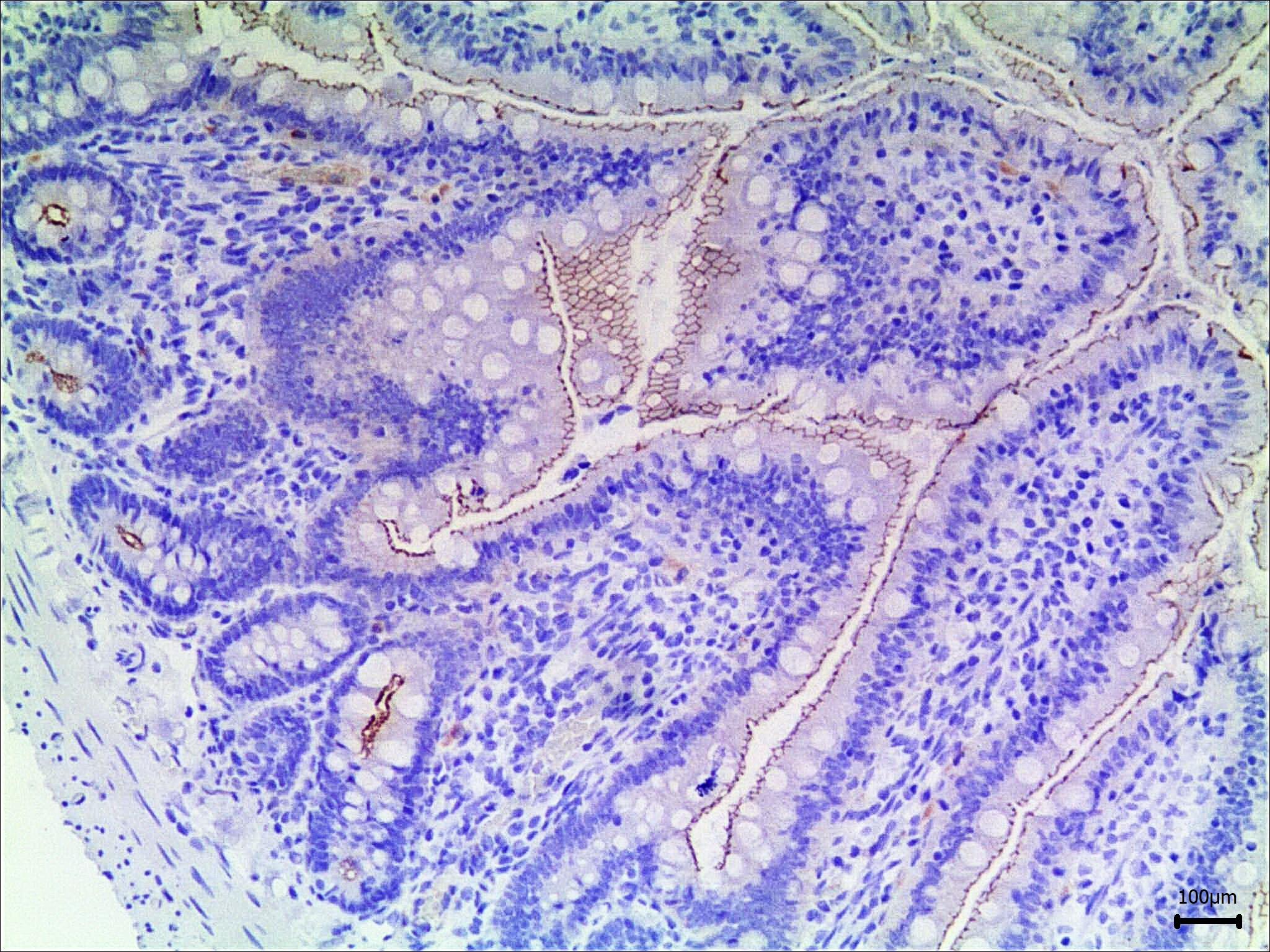

Supplement: Supplementary file 8 [file datasheet8.zip › PTFC Figures-IHC2/zo-1-PTFC.jpg]

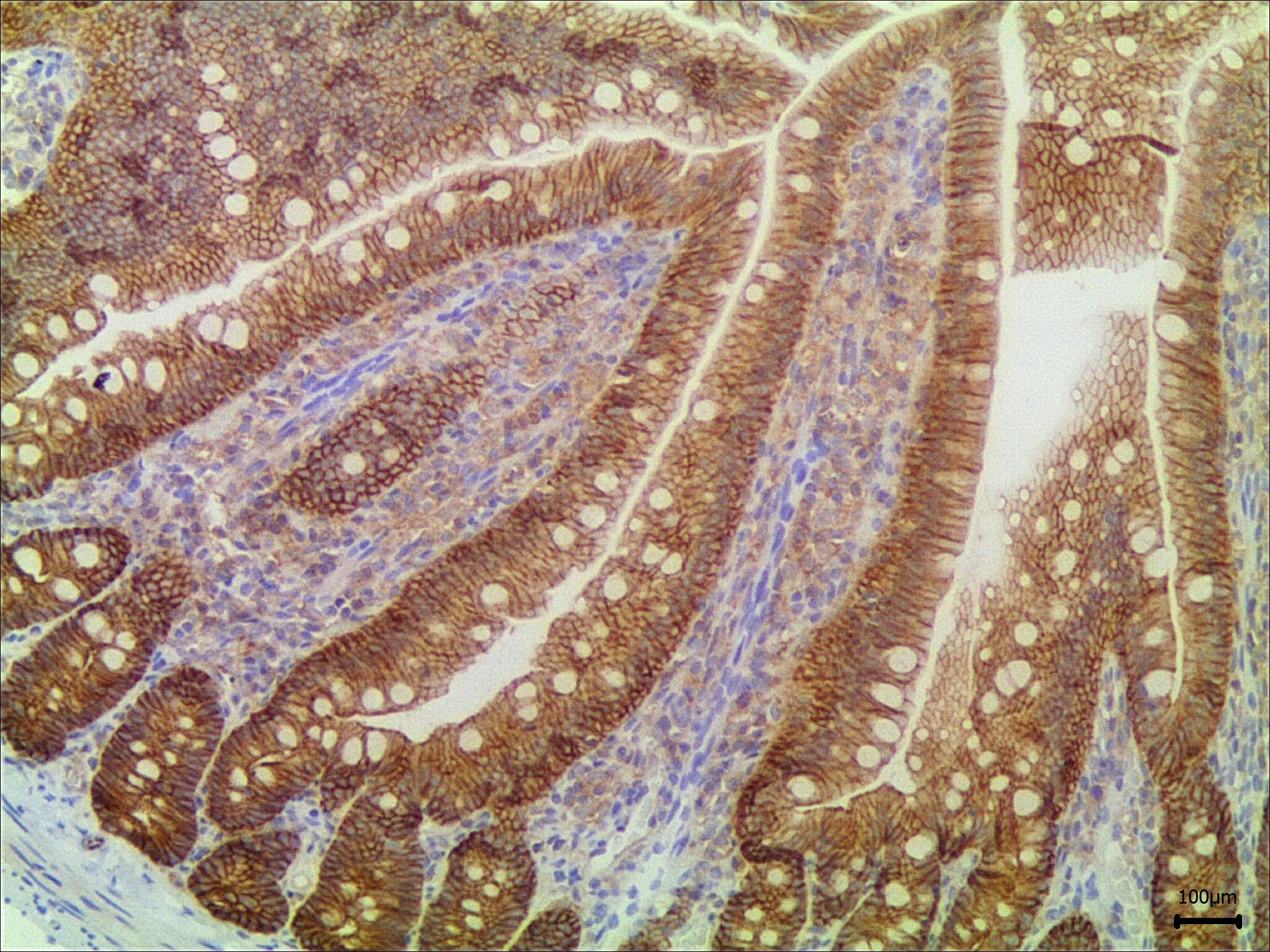

Supplement: Supplementary file 8 [file datasheet8.zip › PTFC Figures-IHC2/occludin-control.jpg]

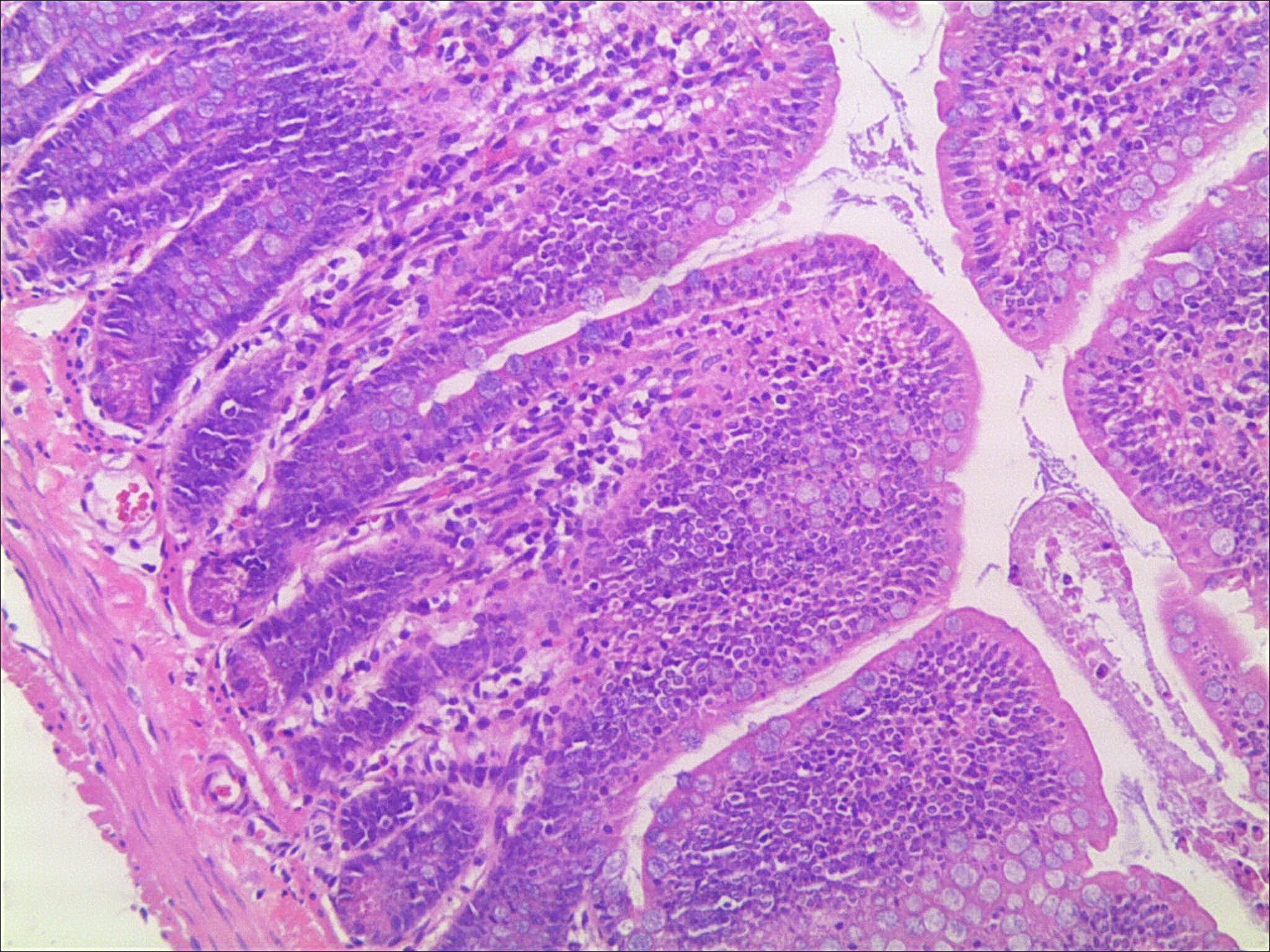

Supplement: Supplementary file 9 [file datasheet9.zip › PTFC SCI Figures-HE Staining/PTFC.jpg]

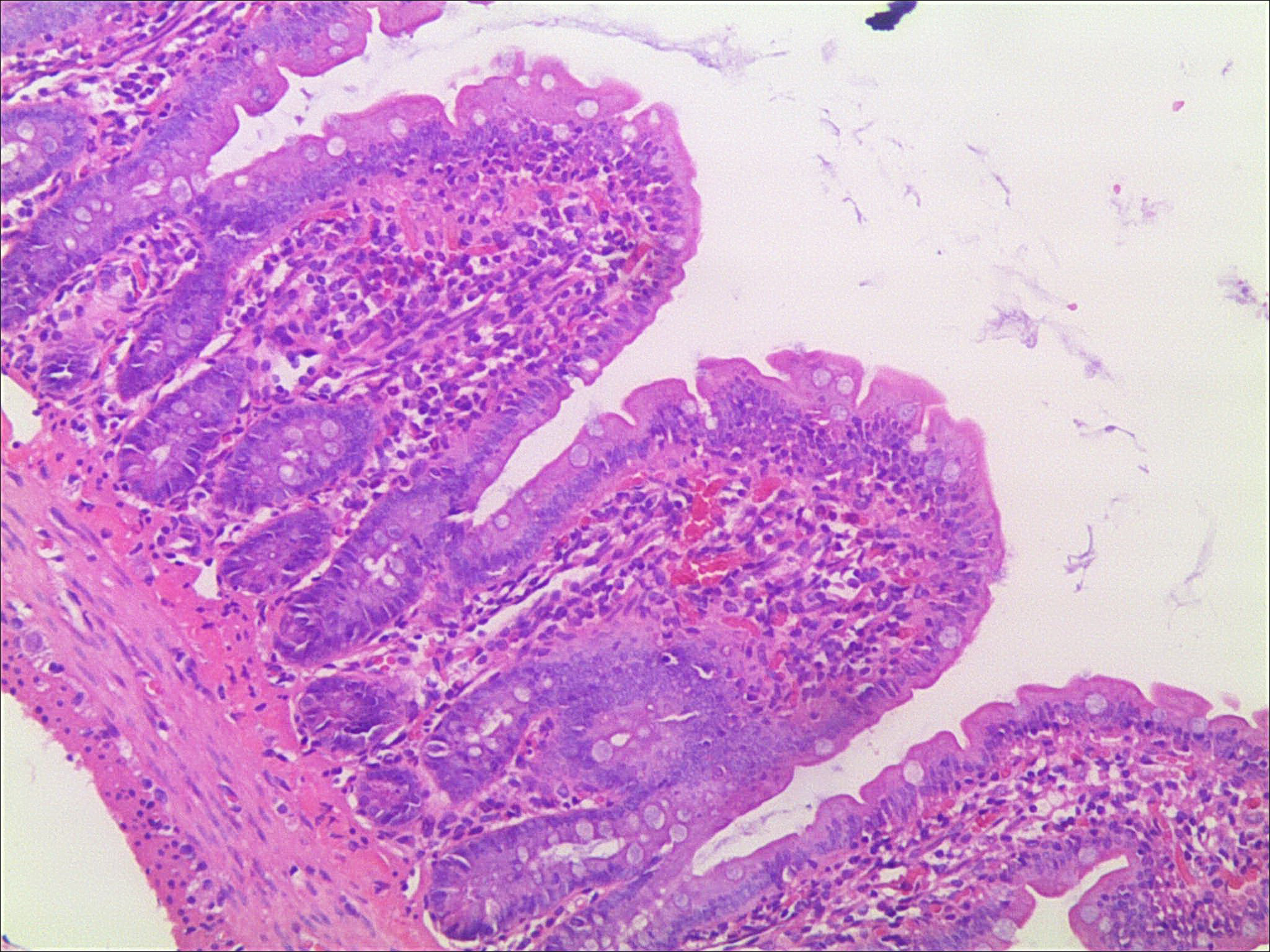

Supplement: Supplementary file 9 [file datasheet9.zip › PTFC SCI Figures-HE Staining/NSAIDs.jpg]

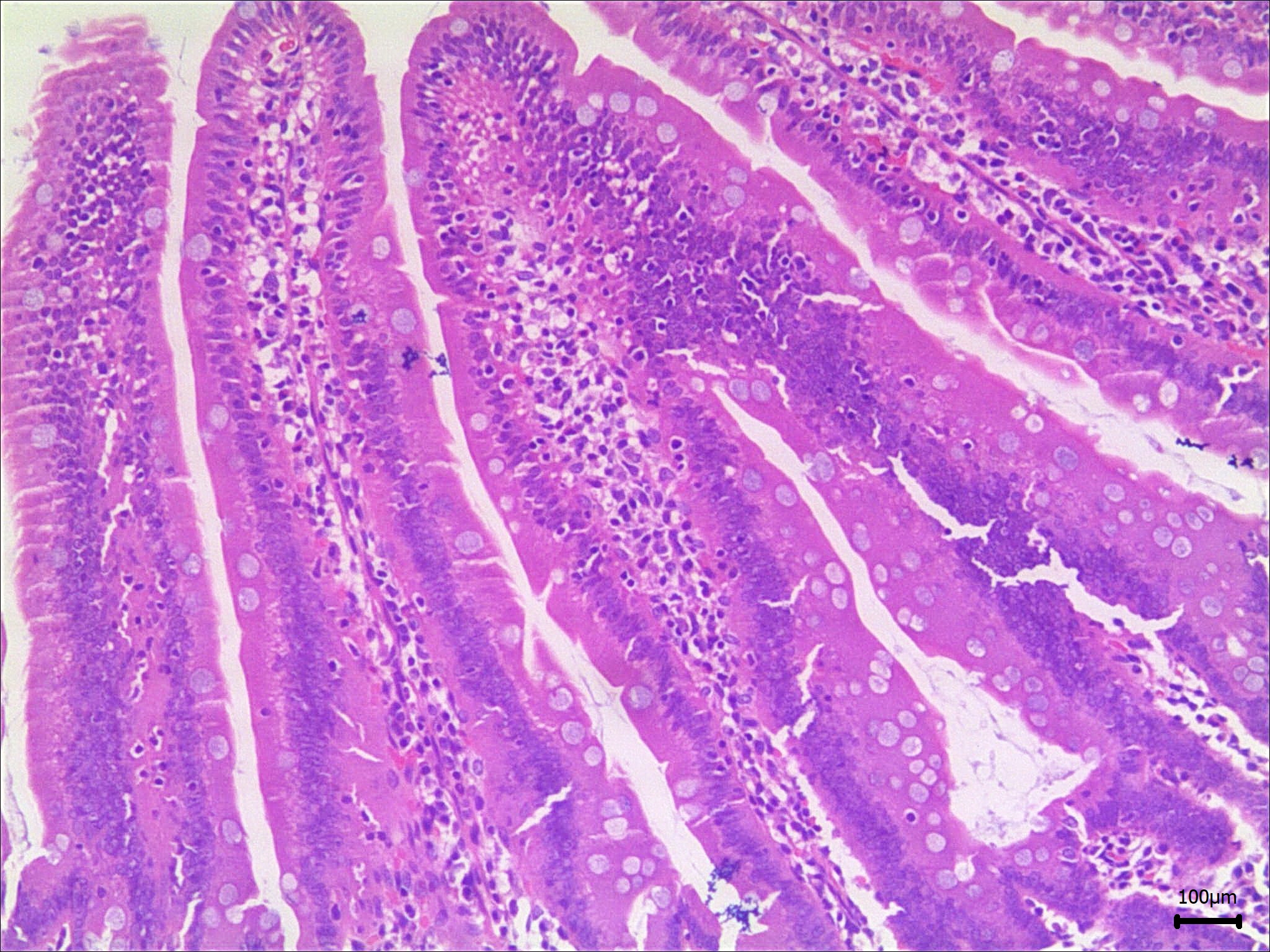

Supplement: Supplementary file 9 [file datasheet9.zip › PTFC SCI Figures-HE Staining/Control.jpg]
